# Cohort Randomised Controlled Trial of a Multifaceted Podiatry Intervention for the Prevention of Falls in Older People (The REFORM Trial)

**DOI:** 10.1371/journal.pone.0168712

**Published:** 2017-01-20

**Authors:** Sarah Cockayne, Joy Adamson, Arabella Clarke, Belen Corbacho, Caroline Fairhurst, Lorraine Green, Catherine E. Hewitt, Kate Hicks, Anne-Maree Kenan, Sarah E. Lamb, Caroline McIntosh, Hylton B. Menz, Anthony C. Redmond, Zoe Richardson, Sara Rodgers, Wesley Vernon, Judith Watson, David J. Torgerson, on behalf of the REFORM study

**Affiliations:** 1 York Trials Unit, Department of Health Sciences, University of York, York, United Kingdom; 2 NIHR Leeds Musculoskeletal Biomedical Research Unit, Chapel Allerton Hospital, Leeds, United Kingdom; 3 Nuffield Department of Orthopaedics, Rheumatology and Musculoskeletal Sciences, Kadoorie Critical Care Research Centre, John Radcliffe Hospital, University of Oxford, Oxford, United Kingdom; 4 National University of Ireland, Galway, Republic of Ireland; 5 School of Allied Health, College of Science, Health and Engineering, La Trobe University, Bundoora, Victoria, Australia; 6 Podiatry Services, Sheffield Teaching Hospitals NHS Foundation Trust, Jordanthorpe Health Centre, Sheffield, United Kingdom; University of Glasgow, UNITED KINGDOM

## Abstract

**Background:**

Falls are a major cause of morbidity among older people. A multifaceted podiatry intervention may reduce the risk of falling. This study evaluated such an intervention.

**Design:**

Pragmatic cohort randomised controlled trial in England and Ireland. 1010 participants were randomised (493 to the Intervention group and 517 to Usual Care) to either: a podiatry intervention, including foot and ankle exercises, foot orthoses and, if required, new footwear, and a falls prevention leaflet or usual podiatry treatment plus a falls prevention leaflet. The primary outcome was the incidence rate of self-reported falls per participant in the 12 months following randomisation. Secondary outcomes included: proportion of fallers and those reporting multiple falls, time to first fall, fear of falling, Frenchay Activities Index, Geriatric Depression Scale, foot pain, health related quality of life, and cost-effectiveness.

**Results:**

In the primary analysis were 484 (98.2%) intervention and 507 (98.1%) control participants. There was a small, non statistically significant reduction in the incidence rate of falls in the intervention group (adjusted incidence rate ratio 0.88, 95% CI 0.73 to 1.05, p = 0.16). The proportion of participants experiencing a fall was lower (49.7 vs 54.9%, adjusted odds ratio 0.78, 95% CI 0.60 to 1.00, p = 0.05) as was the proportion experiencing two or more falls (27.6% vs 34.6%, adjusted odds ratio 0.69, 95% CI 0.52 to 0.90, p = 0.01). There was an increase (p = 0.02) in foot pain for the intervention group. There were no statistically significant differences in other outcomes. The intervention was more costly but marginally more beneficial in terms of health-related quality of life (mean quality adjusted life year (QALY) difference 0.0129, 95% CI -0.0050 to 0.0314) and had a 65% probability of being cost-effective at a threshold of £30,000 per QALY gained.

**Conclusion:**

There was a small reduction in falls. The intervention may be cost-effective.

**Trial Registration:**

ISRCTN ISRCTN68240461

## Introduction

Falls are a major source of morbidity and cost to society [1]. Approximately 30% of people over the age of 65 years living in the community will have a fall each year [2,3]. A fifth of all falls are serious and require medical attention with 5% of falls leading to a fracture.[3] Foot problems may increase the risk of falls with cohort studies indicating a relationship between foot and ankle problems and risk of falling [4,5]. In addition, inappropriate footwear may also contribute to poor balance and an increased risk of falls [6]. A randomised controlled trial (RCT) among 305 community dwelling older people in Australia who had foot pain showed that there was a 36% statistically significant reduction in the rate of falls for people who had received a multifaceted podiatry intervention, comprising of foot and ankle exercises, foot orthoses, footwear advice, subsidy for new footwear, and a falls prevention booklet combined with routine podiatry care compared with those just receiving routine podiatry [7]. In this paper we describe the REFORM (REducing Falls with ORthoses and a Multifaceted podiatry intervention) trial which is a RCT of a podiatric intervention aimed at reducing the incidence of falls among people at high risk of falling.

## Methods

### Study design

We conducted a pragmatic open two-arm cohort randomised controlled trial [8]. Using this design, participants were consented and recruited to an observational cohort study. At recruitment participants were told about the possibility, at some future date, of being offered a package of podiatry care aimed at preventing falls. Those that expressed an interest in the intervention became potentially eligible for randomisation into the embedded RCT. This novel design was chosen in contrast to a ‘standard’ trial design to reduce problems of outcome attrition (by incorporating an outcome assessment run-in period) and possible resentful demoralisation as the control participants are not directly aware of the point of randomisation.

### Eligibility and recruitment

We recruited community dwelling men and women aged 65 years and over from National Health Service (NHS) podiatry clinics in England and from one podiatry clinic at the National University of Ireland, Galway. Detailed methods have been reported elsewhere and the study protocol has been published [9]. In summary, we sent a letter of invitation and screening form to patients who were registered with participating podiatry clinics and who had attended routine podiatry services within the past six months. Patients were asked if they were willing to take part in an observational cohort study (and provide written consent) with the possibility of being offered an additional podiatry intervention to reduce their risk of falling. Eligible, consenting participants were entered into the cohort, and sent a pack of falls calendars on which to record falls and return monthly to the York Trials Unit (YTU). Exclusion criteria included: having neuropathy; dementia or other neurological conditions such as Parkinson’s disease; being unable to walk more than 10 metres without assistance; having a lower limb amputation; and being unwilling or unable to attend the podiatry clinic. To be eligible for randomisation to the intervention patients had to: be willing to accept the intervention; have had a fall within the last 12 months or an injurious fall in the last 24 months; be community dwelling; and have returned at least one monthly falls calendar during their observational run-in phase of the study. Patients who fulfilled all the eligibility criteria, except having had a recent fall, were retained in the cohort and randomised at a later date if they reported a fall. Additionally, during the trial we found that participants who reported an elevated fear of falling in their screening questionnaire were at a similarly increased risk as those with a history of falls. Consequently, we changed our inclusion criteria so such patients could also be randomised. The study was approved by the East of England National Research Ethics Committee (Cambridge East) on 9^th^ November 2011.

### Randomisation

Randomisation was carried out by the York Trials Unit (YTU) secure, remote computer randomisation service. Trial sites informed the YTU of their treatment appointment availability, and then we used block randomisation to allocate participants, with large blocks the size of which was determined by the availability of podiatry appointments and the number of participants eligible to be randomised from that site. For example if there were five podiatry appointments available for a given week then a block of 10 would be used with five allocated to the intervention and five to the control. Participants were mainly randomised 1:1; however, where sites had the capacity to see more or less than half the block size, an appropriate alternative allocation ratio was used. Prediction of allocated group by clinicians was not possible due to the dynamic nature of the randomisation and the use of a remote service; thus allocation concealment was maintained. Once participants had been randomised, those allocated to the intervention group were sent a letter informing them of their group allocation and that the podiatry clinic would be in contact to arrange a trial appointment. Participants allocated to the usual care group were not informed of their group allocation in order to minimise potential attrition and the possibility of resentful demoralisation.

### Intervention group

The multifaceted podiatry intervention comprised of routine podiatry care as determined by the podiatrist and a falls prevention leaflet (both of which the control group also received) in addition to: footwear advice and provision if current footwear was judged to be inappropriate (new footwear supplied by Hotter Footwear^®^ and DB Shoes Ltd); foot orthoses (The x-line^®^, Healthystep, Mossley, UK); and a 30 minutes a day, three times a week home-based foot and ankle exercise programme supplemented with a DVD and explanatory booklet [7]. The elements of the intervention were prescribed to the intervention participants according to the clinical judgement of the podiatrists, all of whom were already employed by the National Health Service and had been given additional training before the start of the trial. For some participants their intervention treatment may have been delivered by a different podiatrist than usual if their routine podiatrist had not received any training. The trial protocol advised that participants be invited to attend one podiatry appointment soon after randomisation and another two to four weeks later. Further appointments (face-to-face or telephone) could be offered if required in addition to the participant’s routine podiatry care. All intervention podiatry time was in addition to routine podiatry time. Further intervention details are provided in the supplementary appendix (S1 File: **REFORM protocol version 5.0 June 2014**).

### Control group

The control participants were not directly informed when they were randomised and the podiatrists were unaware of who the control participants were. Control participants received routine podiatry treatment from their regular podiatrist. Routine podiatry care is prescriptive and dependant on the reason for referral. Typically, routine care includes treatment for painful skin lesions, such as corns and callus, and pathological nails. If footwear is the cause of these pathologies, this will be addressed with footwear advice and possibly simple foot orthoses. Patients are not routinely referred to a podiatrist to address and management risk of falls, and although some sites have recognised the valuable role podiatrists have in addressing this, it is not considered or carried out as part of routine care. Functional insoles (as prescribed for REFORM) could be issued in routine care but would usually be prescribed with more complex modifications via specialised musculoskeletal (MSK) podiatrists. Foot and ankle exercises may also be prescribed but these are usually focussed on resolving a particular foot pathology or injured structure. From the initial training of podiatrists in the trial, it was apparent that exercises were not consistently and/or routinely prescribed and certainly not as a set programme as with the REFORM intervention. As with routine care, it is not typical for MSK podiatrists to prescribe insoles and exercises for the purpose of falls prevention. Therefore, even if participants had seen an MSK podiatrist prior to enrolment in the trial, the intervention for REFORM provided greater focus and diversity to any previous care.

### Outcomes

The pre-specified primary outcome [9] was the incidence rate of falls per participant in the 12 months following randomisation where a fall was defined as “an unexpected event in which the participant comes to rest on the ground, floor, or lower level” [10]. Data were collected via self-reported monthly falls calendars. Participants were asked to record for each day whether or not a fall had occurred. Participants who did not return their calendar were telephoned the week after the calendar was due to be returned to remind them to return the calendar or to collect the data over the telephone, or were sent a reminder letter. Participants were also given a Freephone telephone number to report any incident falls. When a fall was reported the participant was contacted, by telephone, to obtain information about the circumstances of the fall, footwear and orthotic use, and a description of any injuries sustained as well as any medical consequences (e.g. hospital admission). Postal questionnaires were sent to participants at six and 12 months after randomisation to collect health related quality of life (EQ-5D [11]), resource use data, and other pre-specified secondary outcome measures: a single item fear of falling question (“During the past 4 weeks have you worried about having a fall” with six response categories: All of the time, Most of the time, A good bit of the time, Some of the time, A little of the time, None of the time),[9] the Short Falls Efficacy scale–International (Short FES-I), [12] activities of daily living as measured by the Frenchay Activities Index (FAI), [13] depressive symptoms as measured by the Geriatric Depression Scale (GDS), [14] and foot pain severity measured with a 100mm visual analogue scale (VAS). Other secondary outcomes included the proportion of single, and multiple, fallers, time to first fall, and the proportion of participants suffering a fracture as a result of a fall. Since all outcomes in this open trial were participant-reported, it was not possible to conduct blinded outcome assessments.

Details of any adverse events reported to the YTU directly by the participant, a member of their family, or by a member of the research team at the recruiting site were recorded. Any serious adverse events (SAEs) judged to have been related and unexpected were reported to the Research Ethics Committee (REC). Expected events included: deaths; falls; aches and pains in the lower limb lasting for less than 48 hours; new callus/corn formation, blisters, or ulcers; and skin irritation/injury including pressure sores and soft tissue injury.

### Sample size

We powered the study to detect a 10% absolute reduction in the proportion of people having one or more falls over 12 months. We assumed that 50% of the control group would experience a fall. To reduce this to 40% with 80% power (α = 0.05) required 890 participants (445 in each group) allowing for 10% attrition. We chose to power the study using a difference in proportions rather than fall incidence as the latter is not straight forward but this approach should generally be a more conservative estimate of the power of the study to show a difference in falls incidence.

### Statistical analysis

Analyses were conducted using Stata v13 on an available case, modified intention-to-treat (ITT) basis using a two-sided statistical significance level of 0.05, unless otherwise stated. The incidence rate of falls was analysed using a mixed-effects negative binomial regression model controlling for gender, age, and history of falling, with centre as a random effect to account for this potential clustering. The model took account of the different observation periods for each individual by including a variable for the number of months that the participant returned a monthly falls calendar. Coefficients are presented as incidence rate ratios (IRR) with 95% confidence intervals (CI) and p-values. A Complier Average Causal Effect (CACE) analysis [15] to assess the impact of compliance with the intervention on the treatment estimate was undertaken for the primary analysis. Compliance was defined as attending at least one trial appointment. We also considered podiatrist effects by assigning all participants with a podiatrist (actual or counterfactual) and replacing centre with podiatrist as the random effect in the primary analysis model.

The regression models described below to analyse the secondary outcomes were all adjusted as for the primary analysis model (gender, age and history of falling, with centre as a random effect) unless otherwise stated. Mixed logistic regression was used to compare, between the two groups, the proportion of: (i) fallers; (ii) participants who fell 2 or more times; (iii) participants reporting a fracture resulting from a fall; and (iv) of depressed people at 12 months (i.e., those with a total GDS score of six or more). The time from randomisation to first fall was analysed by Cox proportional hazard regression (with shared centre frailty effects). The proportional hazard assumption was evaluated using Schoenfeld residuals. The median time to the first fall and its associated 95% CI was estimated. Continuous outcomes (fear of falling in the past four weeks, Short FES-I, FAI and GDS) were analysed using a covariance pattern model incorporating data from the six and 12 month questionnaires adjusting for baseline score, gender, age, history of falling, treatment group, time and a treatment group-by-time interaction term, with centre as a random effect. Foot pain at 12 months was analysed using a linear mixed model in an ITT analysis, and also in a CACE analysis.

We estimated the costs per participant from an NHS and societal perspective. Our primary economic analysis used multiple imputation because of the high prevalence of missing data. We calculated a cost per quality-adjusted life year (QALY).

### Role of funding source

This project was funded by the National Institute of Health Research (Health Technology Assessment programme) project number 09/77/01. The University of York acted as sponsor. The funder played no role in finalising the study design, data collection, data analysis, data interpretation or report writing. The corresponding author has full access to all study data.

### Protocol changes

After commencement of the study we made the following changes to the study design in addition to clarification of the protocol and adding more recruitment sites. Originally we planned to recruit participants aged 70 years and over but at the beginning of 2013 this was changed to include participants aged 65 years and above (to enhance recruitment). Additionally, other inclusion criteria were modified or clarified (i.e., including patients who had had amputations up to metatarsals, excluding only patients who were unable to walk household distances without aids, including patients using ¾ shoe orthotics). In June 2014 we changed the inclusion criteria to include those patients who had a fear of falling but had not yet fallen.

## Results

**Fig 1:**
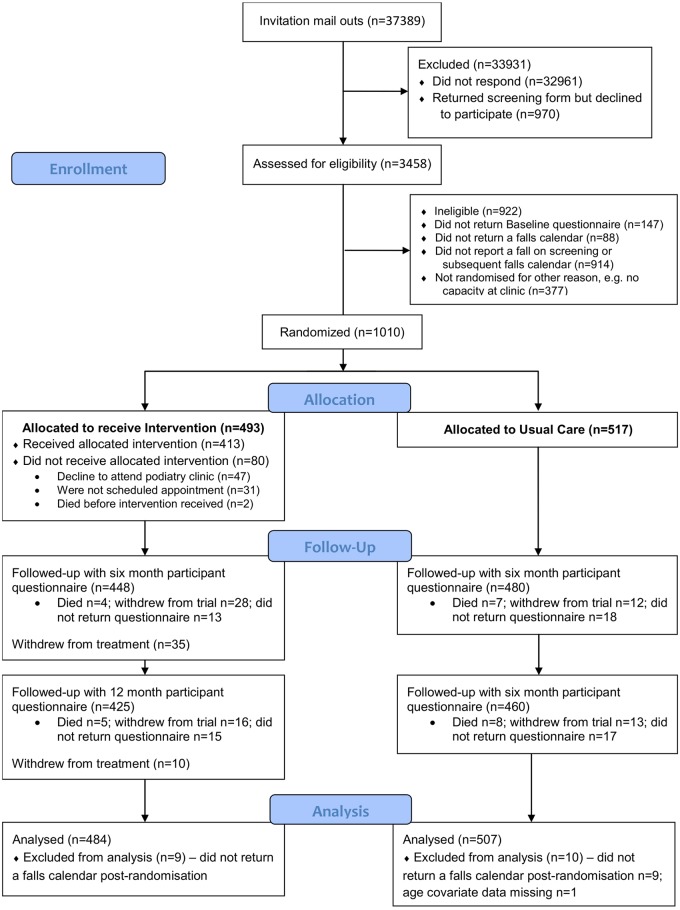
CONSORT flow diagram of participants in the REFORM study.

Between October 2012 and August 2014 we mailed out to 37,389 patients registered at 42 podiatry clinics (Fig 1). Two thousand three hundred and one participants were enrolled into the observational cohort, and of these 1,010 participants were randomised into the trial (493 to the intervention group and 517 to usual care). Table 1 describes the baseline characteristics of the participants by randomised and analysed (in the primary analysis model) groups, which shows that these were balanced between the groups.

**Table 1:** Baseline characteristics of randomised participants by randomised and analysed group (n = 1,010 and 991).

Characteristic	Intervention (randomised, n = 493)	Intervention (analysed, n = 484)	Usual Care (randomised, n = 517)	Usual Care (analysed, n = 507)
**Age, years (mean SD)**	78.1 (7.2)	78.1 (7.2)	77.7 (7.0)	77.6 (7.0)
**Gender, n (%)** *Male*	190 (38.5)	189 (39.1)	210 (40.6)	207 (40.8)
*Female*	303 (61.5)	295 (61.0)	307 (59.4)	300 (59.2)
**Body Mass Index (mean SD)**	27.6 (5.3)	27.6 (5.3)	27.7 (5.4)	27.7 (5.4)
**Ethnic group (White British), n (%)**	492 (99.8)	483 (99.8)	510 (98.7)	500 (98.6)
**Self-reported arthritis, n (%)**	292 (59.2)	286 (59.1)	300 (58)	290 (57.2)
**Live alone, n (%)**	236 (47.9)	230 (47.5)	220 (42.6)	214 (42.2)
**>4 prescribed medications, n (%)**	313 (63.5)	305 (63.0)	304 (58.8)	297 (58.6)
**Current use of foot orthoses, n (%)**	191 (38.7)	189 (39.1)	163 (31.5)	161 (31.8)
**1+ falls in previous 12 months, n (%)**	325 (65.9)	319 (65.9)	332 (64.2)	323 (63.7)
**No fear of falling, n (%)**	130 (26.4)	130 (26.9)	142 (27.6)	140 (27.7)
**Fracture in previous 12 months, n (%)**	38 (7.7)	36 (7.4)	27 (5.2)	27 (5.3)

Four hundred and thirteen intervention participants (83.8%) attended at least one trial podiatry appointment (38 attended only one appointment, and 375 attended two or more). Of the remaining 80, two died close to the time of randomisation, 47 were offered but declined an appointment, and no appointment was made for 31. Participants received a median of two podiatry appointments each (range one to seven). The first appointment occurred a median of 22 days after randomisation (range three to 275 days); and the second appointment a median of 20 days after the first (range six to 343 days). The intervention was delivered by 28 podiatrists who saw a median of ten participants each (range two to 83).

New footwear was provided for 260 intervention participants and an orthotic insole was fitted for 241 participants. The foot and ankle exercises were prescribed where the podiatrist thought it was safe and appropriate to do so, and clinical judgement was used in the advised type and frequency. Data on the exercise equipment prescribed at the initial trial appointment was available for 380 (92.0% of 413) participants; 355 (93.4%) were prescribed a therapy ball, and 358 (94.2%) a resistive band with which to conduct foot and ankle exercises. At 12 months, 66.4% (n = 142) of the 214 participants who received an orthosis and responded to this question reported wearing their orthosis most or all of the time with 85.0% at least some of the time, and 28.9% (n = 101) of the 349 participants who attended at least one trial appointment and responded to this question reported performing the exercises at least three times a week and 74.5% at least once a week.

At least one falls calendar following randomisation was received from 992 (98.2%) participants (intervention n = 484 (98.2%); usual care n = 508 (98.3%)) with 762 participants (75.5%) returning all 12 months’ worth of calendars (intervention n = 360 (73.0%); usual care n = 402 (77.8%)). A total of 1,423 falls were reported: 661 in the intervention group (median 1, range 0 to 23) over a median 365 days (range 6 to 365 days); and 762 in the usual care group (median 1, range 0 to 28) over a median 365 days (range 27 to 365 days). Information on the cause and location was available for 1,172 (82.7%) falls (intervention n = 549 (83.3%); usual care n = 623 (82.1%). Over a third of the falls were caused by a trip (n = 457, 39.0%), and an injury was sustained in over half the falls (n = 655, 55.9%). These injuries include 31 broken bones (from 17 falls in the intervention group and 14 in usual care). The most common bones broken in a fall were the hip or bones in the hand (n = 5 each).

For the primary analysis (Table 2) the adjusted negative binomial model indicated a non-statistically significant 12% reduction in the fall rate in the intervention group relative to usual care (IRR 0.88, 95% CI 0.73 to 1.05, p = 0.16). A sensitivity analysis adjusting the primary model, additionally, for the chance imbalances in prior insole use and the Frenchay Activities Index score at baseline did not materially change the results (IRR 0.88, 95% CI 0.72 to 1.06, p = 0.18). When non-compliance with the intervention was accounted for using a CACE analysis approach, the intervention was seen to have a marginally greater benefit than in the ITT analysis (IRR 0.86, 95% CI 0.69 to 1.06, p = 0.16). Repeating the primary analysis with podiatrist as a random effect in the place of centre had a negligible effect (i.e. beyond two decimal places) on the treatment effect estimate (IRR 0.88, 95% CI 0.73 to 1.05, p = 0.16).

**Table 2:** Falls rates, proportion of fallers and multiple fallers, and quality of life outcomes by treatment group.

Characteristic	Intervention (n = 493)	Usual care (n = 517)	Adjusted treatment effect estimate (95% CI)	p-value
Number of falls per person,				
*mean (min*, *max)*	1.37 (0, 23)	1.50 (0, 28)	IRR 0.88 (0.73 to 1.05)	0.16
1+ falls, *n (%)*	245 (49.7)	284 (54.9)	OR 0.78 (0.60 to 1.00)	0.05
2+ falls, *n (%)*	136 (27.6)	179 (34.6)	OR 0.69 (0.52 to 0.90)	0.01
Time to first fall, days,				
*median (95% CI)*	257 (209, 319)	314 (267, -)^8^	HR 0.88 (0.74 to 1.04)	0.13
Fractures, *n (%)*				
Total number of fractures	19	14	-	
No. participants with fracture	17	14	OR 1.21 (0.59 to 2.49)	0.6
Total number of hip fractures	5	2	-	
Fear of falling^1^, *n*, *mean (SD)*				
Six months	424, 4.4 (1.4)	461, 4.4 (1.3)	AMD 0.08 (-0.05 to 0.21)	0.24
12 months	471, 4.4 (1.4)	453, 4.3 (1.3)	AMD 0.13 (-0.01 to 0.27)	0.07
Short Falls Efficacy Scale^2^,				
*n*, *mean (SD)*				
Six months	425, 12.4 (4.9)	451, 12.2 (4.3)	AMD 0.13 (-0.30 to 0.56)	0.56
12 months	410, 12.7 (4.9)	447, 12.2 (4.4)	AMD 0.30 (-0.14 to 0.73)	0.19
Frenchay Activities Index^3^,				
*n*, *mean (SD)*				
Six months	365, 45.2 (8.3)	405, 45.9 (7.9)	AMD -0.22 (-0.84 to 0.41)	0.5
12 months	372, 45.3 (8.0)	388, 45.8 (8.0)	AMD 0.01 (-0.65 to 0.67)	0.98
Geriatric Depression Scale^4^,				
*n*, *mean (SD)*				
Six months	439, 3.8 (3.2)	467, 3.6 (3.0)	AMD 0.05 (-0.21 to 0.32)	0.7
12 months	418, 3.7 (3.3)	450, 3.4 (3.0)	AMD 0.22 (-0.07 to 0.51)	0.13
Depressed^5^, *total N*, *n (%)*				
Six months	439, 113 (25.7)	467, 101 (21.6)	OR 1.24 (0.91 to 1.69)	0.18
12 months	418, 97 (23.2)	450, 86 (19.1)	OR 1.26 (0.91 to 1.75)	0.16
Foot pain^6^, *n*, *mean (SD)*				
12 months	377, 3.1 (2.8)	426, 2.6 (2.6)	AMD 0.43 (0.06 to 0.80)	0.02
EQ-5D^7^, *n*, *mean (SD)*				
Six months	426, 0.65 (0.2)	455, 0.65 (0.2)	AMD 0.01 (-0.01 to 0.04)	0.39
12 months	414, 0.66 (0.2)	455, 0.66 (0.2)	AMD 0.01 (-0.01 to 0.04)	0.29

^1^“During the past four weeks have you worried about having a fall?” response categories: 1 = all of the time; 2 = most of the time; 3 = a good bit of the time; 4 = some of the time; 5 = a little of the time; and 6 = none of the time;

^2^scored 7–28 lower score indicates less concern about falling;

^3^scored 15–60 higher score indicates greater activity;

^4^scored 0–15 higher score indicates greater depression;

^5^as indicated by score of 6 or more on GDS;

^6^0 (no pain) to 10 (worst possible pain);

^7^scored 0–1 where 1 indicates best imaginable health state.

IRR = incidence rate ratio; OR = odds ratio; HR = hazard ratio; AMD = adjusted mean difference;

^8^upper limit not calculable.

In a post-hoc analysis of the primary outcome we found no interaction (interaction term p = 0.93) between treatment effect and gender (males IRR 0.87, 95% CI 0.64 to 1.17; females 0.86, 95% CI 0.68 to 1.09).

**Fig 2:**
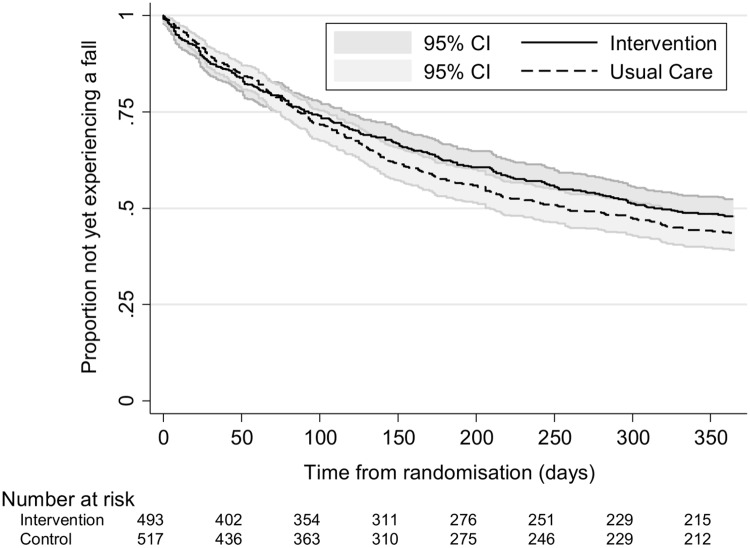
Kaplan-Meier curves by randomised group for time to first fall.

Fewer participants in the intervention group had one or more falls (n = 245 (49.7%) vs. n = 284 (54.9%) usual care participants; adjusted odds ratio (OR) 0.78, 95% CI 0.60 to 1.00, p = 0.05). There was also a lower proportion of participants in the intervention group than the usual care group who reported two or more falls on their falls calendars following randomisation (n = 136 (27.6%) vs. n = 179 (34.6%); adjusted OR 0.69, 95% CI 0.52 to 0.90, p = 0.01). The median time to the first fall was estimated at 314 days (95% CI 267, upper limit not calculable) in the intervention group and 257 days in the usual care group (95% CI 209 to 319). Kaplan Meier survival curves are presented for each group in Fig 2. The adjusted hazard ratio from the Cox proportional hazards model for the treatment effect was 0.88 (95% CI 0.74 to 1.04, p = 0.13) indicating that the hazard or chance of falling at any particular time was lower in the intervention group than the usual care group, but this ratio was not statistically significant.

Six and 12 month follow-up data for the secondary outcomes were available for a potential 928 (91.9%) (intervention n = 448 (90.9%); usual care n = 480 (92.8%)) and 885 (87.6%) (intervention n = 425 (86.2%); usual care n = 460 (89.0%)) participants, respectively. No statistically significant differences between the two groups were observed at six or 12 months in the fear of falling question, Short FES-I, FAI, GDS or the proportion of depressed participants (Table 2).

Over the 12 month follow-up, 31 (3.1%) participants (intervention n = 17 (3.4%); usual care n = 14 (2.7%)) reported breaking or fracturing a bone as a result of a fall (adjusted OR 1.21, 95% CI 0.59 to 2.49, p = 0.60). Two participants, both in the intervention group, reported repeated fractures from two different events.

Participants in the intervention group reported greater foot pain at 12 months (mean 31mm (SD 28) vs. 26mm (SD 26) VAS; adjusted mean difference in ITT analysis 4.3, 95% CI 0.6 to 8.0, p = 0.02; and in CACE analysis 5.0, 95% CI 0.8 to 9.2, p = 0.02).

There were 95 reported serious adverse events (SAEs) in the period between randomisation and one month following trial end (i.e., 13 months after randomisation), for 49 (9.9%) participants in the intervention group and 37 (7.2%) participants in usual care (Table 3). The majority of participants (n = 78; 90.7%) reported only one event. Within the reporting period, there were 23 reported deaths (eight in the intervention group and 15 in usual care). For seven deaths, the relationship to research procedures could not be assessed due to a lack of information, but for those that could, none were deemed to be related. Nearly two thirds of all SAEs were hospitalisations (n = 62, 65.3%). Two events considered to be life/limb threatening were in the intervention group and one of these was related to the intervention (pressure ulceration of toe caused by insole).

**Table 3:** Serious Adverse Events by randomised group.

Serious Adverse Events	Intervention	Usual care	Total
	(n = 493)	(n = 517)	(n = 1010)
**Total number of SAEs**	53	42	95
**Number of participants with one or more SAEs**	49	37	86
**Number of events per participant, n (%)**			
*1*	45 (91.8)	33 (89.2)	78 (90.7)
*2*	4 (8.2)	3 (8.1)	7 (8.1)
*3*	0 (0.0)	1 (2.7)	1 (1.2)
**Event details, n (%)**			
*Death*	8 (15.1)	15 (35.7)	23 (24.2)
*Hospital required/prolonged*			
*Life/limb threatening*	36 (67.9)	26 (61.9)	62 (65.3)
*Disability*	2 (3.8)	0 (0.0)	2 (2.1)
*Other*	0 (0.0)	0 (0.0)	0 (0.0)
	7 (13.2)	1 (2.4)	8 (8.4)
**Intensity, n (%)**			
*Mild*	0 (0.0)	0 (0.0)	0 (0.0)
*Moderate*	5 (9.4)	2 (4.8)	7 (7.4)
*Severe*	47 (88.7)	40 (95.2)	87 (91.6)
Missing*	1 (1.9)	0 (0.0)	1 (1.1)
**Outcome, n (%)**			
*Recovered fully*	22 (41.5)	12 (28.6)	34 (35.8)
*Recovered partially*	6 (11.3)	2 (4.8)	8 (8.4)
*On-going*	16 (30.2)	13 (31.0)	29 (30.5)
*Died*	8 (15.1)	15 (35.7)	23 (24.2)
*Missing*	1 (1.9)	0 (0.0)	1 (1.1)
**Relationship to any of the research procedures, n (%)**			
*Unrelated*	35 (66.0)	31 (73.8)	66 (69.5)
*Unlikely*	8 (15.1)	6 (14.3)	14 (14.7)
*Possibly*	3 (5.7)	0 (0.0)	3 (3.2)
*Probably*	0 (0.0)	0 (0.0)	0 (0.0)
*Definitely*	2 (3.8)	0 (0.0)	2 (2.1)
*Not able to assess*	4 (7.6)	5 (11.9)	9 (9.5)
*Missing*	1 (1.9)	0 (0.0)	1 (1.1)
**Expectedness, n (%)**			
*Expected*	48 (90.6)	37 (88.1)	85 (89.5)
*Unexpected*	4 (7.6)	5 (11.9)	9 (9.5)
*Missing*	1 (1.9)	0 (0.0)	1 (1.1)

*Event with missing outcome, relationship and expectedness was initially reported on participant’s 12 month questionnaire and followed up by a member of the research team. Participant reported breaking their leg and developing tendinopathy following a falls 8 months earlier. Event was not reported at the time and limited information was available when this event was followed up.

The analysis of the EQ-5D shows that the participants randomised to the intervention experienced, on average, 0.0129 (95% CI -0.0050 to 0.0314) more QALYs over 12 months than the usual care group. However, the intervention was more costly than usual care (on average £252.17 more per participant 95% CI -69.48 to 589.38). When adjusted for all covariates (which include baseline utility) the incremental cost per QALY ranged between £19,494 and £20,593. For both the NHS perspective (primary) and societal perspective (secondary analysis), the probability of the intervention being the more cost-effective option was above 0.60 for the incremental analysis adjusted for baseline EQ-5D, and above 0.65 when incremental QALYs are adjusted for all covariates. The cost per fall averted was £1,254.

## Discussion

The REFORM trial is the largest study of a podiatric programme including a foot and ankle exercise regime to reduce the risk of falling, randomising 1,010 participants. The primary clinical outcome for the trial was the number of falls reported on monthly falls calendars in the 12 months following randomisation. In total, 992 (98.2%) trial participants returned at least one falls calendar following randomisation with similar proportions across the two groups. We found a 12% reduction in the rate of falls per person-year and an absolute reduction of 5% in the number of participants who had one or more falls over the 12 months from randomisation. The difference was not statistically significant in our pre-specified primary outcome of incidence rate of falls; however, the difference in the proportion who had at least one fall or two or more falls, was statistically significant. Time to first fall was reduced in the intervention group but not statistically significantly. The results of the economic evaluation conducted alongside the REFORM trial suggest that the multifaceted intervention could be a cost-effective option for falls prevention with the incremental cost per QALY (based on health related quality of life) ranging between £19,494 and £20,593, which is approximately the threshold that the UK’s National Institute for Health Excellence (NICE) deems as being ‘cost effective’.

Our results to some extent support the earlier findings by Spink and colleagues [5]. In this Australian trial among 305 community dwelling men and women (mean age 74 years) who were suffering from disabling foot pain and had an elevated risk of falling, a reduction in the incidence rate of falls was observed (IRR 0.64, 95% CI 0.45 to 0.91, p = 0.01). The Australian population were similar to ours in that they were all receiving routine podiatry care and were recruited from podiatry patient lists. On the other hand, participants had to be suffering from disabling foot pain, which was not necessarily the case for our population; participants may have had foot pathology but not necessarily significant foot pain. This could be a possible reason for the study differences. Our study population had a higher risk of falling with the usual care group sustaining an average of 1.5 falls per year compared with 1.06 for the Australian patient group. Similarly, 55% of our usual care participants sustained one or more falls compared with 49% in the Spink study. Combining the two studies in an individual patient data meta-analysis showed a reduction in the IRR of falls, which was not statistically significant (IRR = 0.79, 95% CI 0.56 to 1.10, I squared 66.3%). However, the proportion who had one or more falls was statistically significant (OR = 0.79, 95% CI 0.64 to 0.99, I squared 0.0%) as was those who had two or more falls (OR 0.79, 95% CI 0.64 to 0.99, I squared 0.0%).

The key elements of the interventions were similar, comprising of foot and ankle exercises, an orthosis, and an assessment for poor footwear. Both studies were conducted among patients who were receiving ‘standard’ podiatry care, which aimed to maintain nails and reduce painful conditions, such as corns and callus, which are associated with an increased risk of falling. There were some differences, however. We did not use the same orthosis as the Australian study and the foot and ankle exercises were modified, partly in light of lessons learnt from the Australian study. In our study, where possible, new footwear was provided to participants in the intervention group whose own current footwear was inappropriate. Adherence to the trial footwear was mixed. Some participants being very satisfied with the choice and wore their footwear. Others however, did not, for reasons of comfort and footwear being the wrong size. In the Australian trial, participants were provided with subsidy for new footwear in the form of a voucher.

This was a large pragmatic trial and we used a novel design–a cohort randomised trial–to evaluate this podiatric intervention. The design had several strengths: the use of a run-in period with outcome data collection may have reduced the incidence of post-randomisation attrition; and the usual care group were unaware of the exact time they were randomised which, in theory, should have limited resentful demoralisation. The design also allowed us to recruit participants who were initially ineligible due to absence of a fall but later became eligible once they had fallen when part of the observational cohort. A limitation of the study is that the participants were recruited from podiatry clinics so the estimated impact of the intervention among people who do not see a regular NHS podiatrist or receive care from a private podiatrist may be different. There is possibly some ‘dilution’ effect as some intervention podiatrists were also seeing control patients and may have given falls prevention advice to members of the control group. We think that any dilution is likely to have been trivial as the full intervention required at least two additional treatment sessions. In addition, the podiatrists did not know who the control patients were. Using a run-in period may also have biased the sample towards volunteers with a heightened interest and commitment to the intervention. Furthermore, the intervention is a ‘complex’ one and our design does not allow us to estimate the different contributions of changes in footwear, addition of an orthotic insole, or foot and ankle exercise to the observed effect. It may well be that one or more of the interventions included in the ‘package of care’ may be ineffective. Participants were all recruited from podiatry clinic lists. The reason for this was to ensure that we could identify an additional effect of the intervention not confounded by routine podiatric care. Consequently, the trial cannot answer the question of whether the intervention is effective among patients who do not have routine podiatry care. However, our results suggest that there could be a role for NHS podiatrists to reduce the risk of falling among their patients and that this program could be a cost effective use of podiatric time for patients at high risk of falling.

## Supporting Information

S1 CONSORT Checklist (DOC)

S1 File REFORM protocol version 5.0 June 2014. (DOC)
