# Supplementary material for: Cohort Randomised Controlled Trial of a Multifaceted Podiatry Intervention for the Prevention of Falls in Older People (The REFORM Trial)
Source: PLoS One. 2017 Jan 20;12(1):e0168712. doi: 10.1371/journal.pone.0168712 (PMC5249075; doi:10.1371/journal.pone.0168712)
Supplement: S1 File — (DOC) [file pone.0168712.s002.doc]

REFORM: A randomised trial of a multifaceted podiatry intervention for fall prevention

Trial protocol

| **Authorised by:** | |  |  |  | |  | | | |
| --- | --- | --- | --- | --- | --- | --- | --- | --- | --- |
| Name: Prof David Torgerson |  | |  | Role: |  | | | |  |
| Role: Chief Investigator  Signature: |  | |  |  | | |  | | |
| Date: 16^th^ June 2014  Protocol: Number 5.0  ISRCTN ISRCTN68240461  Sponsor’s protocol number: REFORM12082011 |  | |  |  | | |  | | |
|  | e | |  |  | | |  | | |
|  |  | |  | | | | |  | |

The REFORM trial is funded by: The REFORM trial is sponsored by:


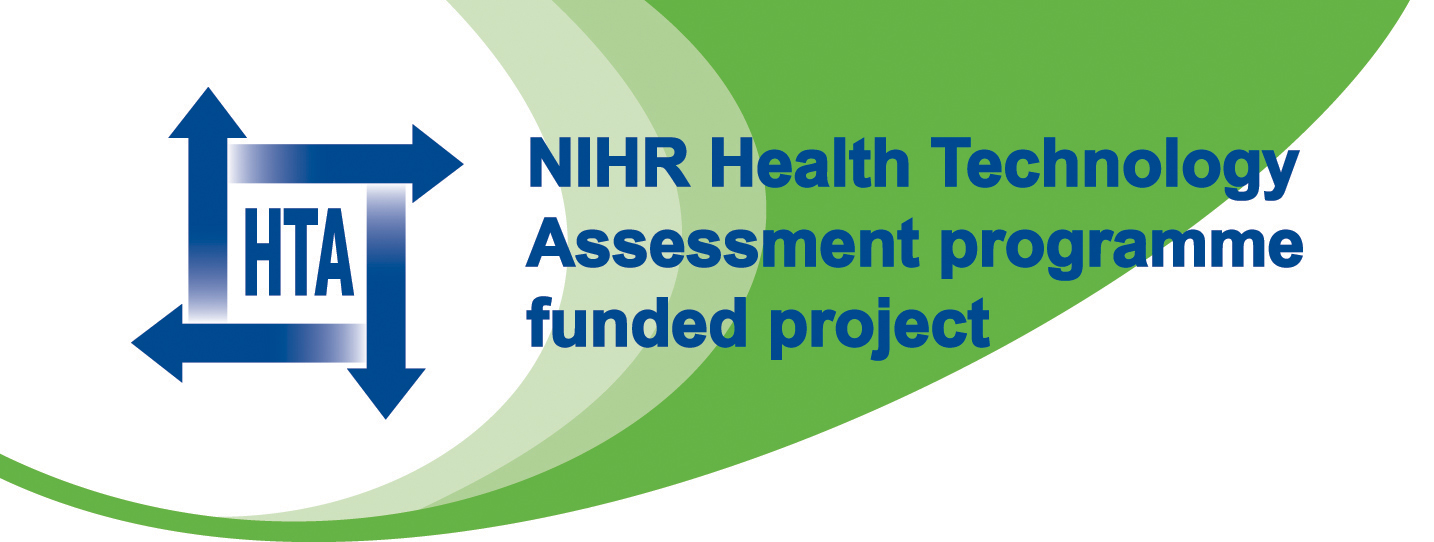

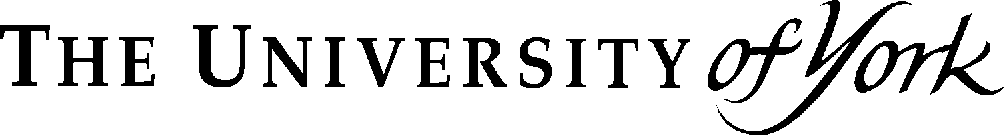


Amendments to protocol

A Protocol version 2.0 22^nd^ January 2013

Clarification to the protocol has been made as follows: Update to research team details; additional recruitment sites included; completion of the consent form; clarification that the qualitative interviews with participants may be conducted over the phone; clarification about method of contacting trial participants regarding data queries; removal of the requirement for patients to return decline forms for the main trial; change in the age criteria for the study from over 70 years to over 65 years of age; clarification of the exclusion criteria to only exclude patients with an amputation at or proximal to the level of the metatarsals and to exclude only patients who are unable to walk household distances without the help of a walking aid such as a Zimmer frame or walker or person to assist. Patients who use a walking stick however, will be eligible for the study. Patients who are currently wearing a full or ¾ length in shoe foot orthotic for the purpose of altering or modifying foot function will no longer be excluded; exercise data will be collected via question sent at 1, 3, 6 and 12 months as opposed to an exercise calendar; clarification to the trial intervention; additional expected adverse events related to the intervention included

B Protocol version 3.0 5^th^ March 2013

Clarification to the protocol has been made as follow: The research podiatrist may give podiatry patients information about the study in the podiatry clinic; where multidisciplinary team exist other healthcare professionals such as occupational therapists, falls practitioners and physiotherapists may support the opportunistic recruitment; clarification to the eligibility criteria that any patient with a lower limb amputation will be excluded from the trial; that participants no longer need to complete 3 falls calendars to be eligible for the study; additional sites added.

C Protocol version 4.0 19^th^ March 2014

Increase in the number of participants recruited to the cohort to 2,600 Additional recruitment sites added.

D Protocol version 5 16^th^ June 2014

Additional inclusion criteria to include those who report a fear of falling on their baseline questionnaire.

CONTENTS TABLE

| **Section** | **Item** | **Page number** |
| --- | --- | --- |
|  |  |  |
| 1 | General information | 5 |
|  |  |  |
| 2 | Summary | 7 |
|  |  |  |
| 3 | Trial identifier | 8 |
| 3.1 | Full title of trial | 8 |
| 3.2 | Acronym | 8 |
| 3.3 | ISRCTN | 8 |
| 3.4 | Funder | 8 |
| 3.5 | Sponsor | 8 |
|  |  |  |
| 4 | Background | 9 |
| 4.1 | Problem to be addressed | 9 |
| 4.2 | The need for a trial | 9 |
| 4.3 | Research objectives | 10 |
|  |  |  |
| 5 | Study design | 11 |
| 5.1 | Recruitment of the REFORM epidemiological cohort | 11 |
| 5.1.1 | Recruitment of sites | 11 |
| 5.1.2 | Identification of patients to receive invitation mail out | 11 |
| 5.1.3 | Patients who wish to decline participation in the REFORM study | 12 |
| 5.1.4 | Participation in other REFORM studies | 12 |
| 5.1.5 | Patients who wish to take part in the REFORM study | 12 |
| 5.2 | The REFORM pilot study | 13 |
| 5.2.1 | Eligibility criteria for the REFORM pilot study | 13 |
| 5.2.2 | Recruitment to the pilot study | 14 |
| 5.2.2.1 | Recruitment and sample size for the pilot study | 14 |
| 5.2.2.2 | Recruitment and sample size for the qualitative study | 15 |
| 5.2.3 | Interventions | 15 |
| 5.2.3.1 | Foot orthotic | 15 |
| 5.2.3.2 | Foot and ankle exercise programme | 15 |
| 5.2.3.3 | Footwear assessment and shoe provision | 17 |
| 5.2.4.1 | Data collection and analysis for quantitative study | 17 |
| 5.2.4.2 | Nested qualitative study | 17 |
| 5.2.4.3 | Qualitative Data analysis | 18 |
| 5.3 | The REFORM trial | 19 |
| 5.3.1 | Trial design | 19 |
| 5.3.2 | Inclusion criteria | 19 |
| 5.3.3 | Exclusion criteria | 19 |
| 5.3.4 | Primary outcome | 20 |
| 5.3.5 | Secondary outcome | 20 |
| 5.3.6 | Sample size | 20 |
| 5.3.7 | Participant withdrawal | 20 |
| 5.3.8 | Randomisation | 21 |
| 5.3.9 | Blinding | 21 |
| 5.3.10 | Intervention group | 21 |
| 5.3.11 | Control group | 24 |
| 5.3.12 | Health economics | 24 |
| 5.3.13 | Adverse events | 26 |
| 5.4 | Data collection and analysis | 24 |
| 5.4.1 | Quantitative data collection | 27 |
| 5.4.2 | Data collection for the primary outcome for the REFORM trial | 28 |
| 5.4.3 | Data collection for secondary outcomes and process data for the REFORM trial | 28 |
| 5.4.4 | Statistical analysis | 29 |
| 5.4.5 | Statistical analysis of the primary outcome | 29 |
| 5.4.6 | Statistical analysis of the secondary outcomes | 29 |
| 5.4.7 | Intervention adherence | 30 |
| 5.5 | Definition of the end of the trial | 31 |
|  |  |  |
| 6 | Trial monitoring | 30 |
| 6.1 | Site monitoring | 31 |
| 6.2 | Standard operating procedures | 31 |
|  |  |  |
| 7 | Ethical issues | 31 |
| 7.1 | Anticipated risks and benefits | 32 |
| 7.2 | Informing participants of anticipated risks and benefits | 32 |
| 7.3 | Obtaining informed consent | 32 |
| 7.4 | Retention of study documentation | 33 |
|  |  |  |
| 8 | Service user involvement | 33 |
|  |  |  |
| 9 | Trial management | 33 |
| 9.1 | Sponsorship | 33 |
| 9.2 | Indemnity | 34 |
| 9.3 | Funding | 34 |
| 9.4 | Independent Steering Committee | 34 |
| 9.5 | Trials management group | 35 |
|  |  |  |
| 10 | Publication policy | 35 |
|  |  |  |

**1 General Information**

Chief Investigator

Prof David Torgerson University of York, Dpt Health Sciences, York Trials Unit, ARRC Building, Lower Ground Floor, York YO10 5DD

Email: [david.torgerson@york.ac.uk](mailto:david.torgerson@york.ac.uk)

Co-applicants

Dr Joy Adamson University of York, Dpt Health Sciences, York Trials Unit, ARRC Building, Lower Ground Floor, York YO10 5DD

Email: [**joy.adamson@york.ac.uk**](mailto:joy.adamson@york.ac.uk)

Mr Robin Hull 37 Monkgate York YO31 7PB

Email [**Robin.Hull@hdft.nhs.uk**](mailto:Robin.Hull@hdft.nhs.uk)

Dr Anne-Maree Keenan NIHR Leeds Musculoskeletal Biomedical Research Unit, Chapel Allerton Hospital Leeds LS4 7SA

Email:**a.keenan@leeds.ac.uk**

Prof Sarah Lamb Warwick Clinical Trials Unit, The University of Warwick Gibbet Hill Road Coventry CV4 7AL

Email: **s.lamb@warwick.ac.uk**

Dr Caroline McIntosh National University of Ireland, room 104, Aras Moyola, Galway Ireland

Email: [**caroline.mcintosh@nuigalway.ie**](mailto:caroline.mcintosh@nuigalway.ie)

Prof Hylton Menz La Trobe University, Musculoskeletal Research Centre, Faculty of Health Sciences, Victoria, Australia

Email: [**h.menz@latrobe.edu.au**](mailto:h.menz@latrobe.edu.au)

Prof Anthony Redmond 2^nd^ Floor, Chapel Allerton Hospital, Harehills Lane Leeds LS7 4SA

Email: [**a.redmond@leeds.ac.uk**](mailto:a.redmond@leeds.ac.uk)

Prof Wesley Vernon Sheffield Community Services, Jordanthorpe Health Centre, 1 Dyche Close Sheffield, S8 8DJ

Email: **wesley.vernon@nhs.net**

Dr Judith Watson University of York, Dpt Health Sciences, York Trials Unit, ARRC Building, 1^st^ Floor, York YO10 5DD

Email: **jude.watson@york.ac.uk**

Dr Catherine Hewitt University of York, Dpt Health Sciences, York Trials Unit, ARRC Building,

Ground Floor, York YO10 5DD

Email **catherine.hewitt@york.ac.uk**

Trial management

Trial Coordinator: Sarah Cockayne

and co-applicant University of York, Dpt Health Sciences, York Trials Unit, ARRC Building, Ground Floor, York YO10 5DD

Email: [**sarah.cockayne@york.ac.uk**](mailto:sarah.cockayne@york.ac.uk)

Tel: 01904 321736 Fax: 01904 321387

Trial statistician Muhammad Usman, University of York, Dpt Health Sciences, York Trials Unit, ARRC Building,

Ground Floor, York YO10 5DD

Email: **muhammad.usman@york.ac.uk**

Tel: 01904 321118

Health Economist: Dr Belen Corbacho, University of York, Dpt Health Sciences, York Trials Unit, ARRC Building,

Ground Floor, York YO10 5DD

Email: **belen.corbacho@york.ac.uk**

Tel: 01904 321852 Fax: 01904 321387

Data manager: Mr Matthew Bailey, University of York, Dpt Health Sciences, York Trials Unit, ARRC Building, Lower Ground Floor, York YO10 5DD

Email: **matthew.bailey**[@**york.ac.uk**](mailto:sarah.cockayne@york.ac.uk)

Tel: 01904 321364 Fax: 01904 321387

Data manager: Mrs Val Wadsworth

University of York, Dpt Health Sciences, York Trials Unit

ARRC Building, Lower Ground Floor, York YO10 5DD

Email: **val.wadsworth@york.ac.uk**

Tel: 01904 32 1363 Fax: 01904 321387

Research Podiatrist Lorraine Loughrey, 2^nd^ Floor, Chapel Allerton Hospital, Harehills Lane Leeds LS7 4SA

Email: **L.R.Loughrey@leeds.ac.uk**

Trial secretary: Mrs Maggie Gowlett

University of York, Dpt Health Sciences, York Trials Unit

ARRC Building, Lower Ground Floor, York YO10 5DD

Email:**maggie.gowlett@york.ac.uk**

Tel: 01904 32 1366

Fax: 01904 321387

**2 SUMMARY**

Falls and fall related fractures are a serious cause of morbidity and cost to society, [1] a burden which will increase with an ageing population. Falls results from an interaction between environmental hazards and a broad array of medical conditions and physiological impairments. [2] There is some evidence to suggest that foot problems, and inappropriate footwear may increase the risk of falls, therefore it has been suggested that podiatry may have a role to play in falls prevention. There have been two relevant Cochrane reviews on falls prevention, neither of which found any trials focusing on podiatry-related interventions. Several studies have suggested that some treatments provided by podiatrists, such as lesion debridement [19] and foot orthoses [20] may improve balance. Apart from one recently completed trial in Australia [23] there has not been a large pragmatic trial evaluating the role of podiatric care combined with footwear advice and orthotic inserts for falls prevention or any assessment of whether it would be economically viable within a UK setting. This trial aims to address these issues.

In this study we will undertake a randomised controlled trial to evaluate the clinical and cost effectiveness of a multifaceted podiatry intervention. The study will randomly allocate 890 participants to receive either a multifaceted podiatry intervention or usual care provided by the podiatrist or GP and a falls prevention leaflet. Data on the number of falls the participant experiences will be collected via patient self-reported falls calendars in the 12 months following randomisation. We will recruit patients through podiatry clinics, GP practices and falls prevention clinics in eighteen different geographical areas: North Yorkshire, Leeds, Sheffield, Hull, Salford, Galway, Republic of Ireland, Doncaster, Grimsby, Barnsley, Sunderland, Norfolk, South Tyneside, North Tees and Hartlepool, Kent Community Health, NHS Solent, Southern Health, Medway Community Trust, South Essex Partnership Trust . If recruitment is slower than anticipated we will consider opening additional sites within the UK.

A nested qualitative study will also be carried out to examine the acceptability of the intervention as a whole package of care to both the trial participants and the podiatry practitioners.

**3 STudy identifiers**

**3.1 Full title of trial**

Randomised trial of a multifaceted podiatry intervention for fall prevention

**3.2 Acronym: REFORM:** REducing Falls with ORthoses and a Multifaceted podiatry intervention

**3.3 ISRCTN:** TBA

**3.4 Funder:** NIHR HTA Programme grant number 09/77/01

**3.5**  **Sponso**r**:** The University of York

**4 Background to the trial**

**4.1 Problem to be addressed**

Falls and fall related fractures are a serious cause of morbidity and cost to society, [1] a burden which will increase with an ageing population. The importance of fall related injuries has been recognised in the recently published National Service Framework (NSF) for Older People. The NSF calls for health improvement plans to be devised that will reduce the burden of fall related injuries.

It is well recognised that falls result from an interaction between environmental hazards and a broad array of medical conditions and physiological impairments. [2] Foot problems, however, may also increase the risk of falls. As foot problems affect one in three community dwelling people over the age of 65 years [3] and are associated with reduced walking speed and difficulty performing activities of daily living [4-6] this presents a further problem with the ageing population.

**4.2 The need for a trial**

There have been two relevant Cochrane reviews on falls prevention, one relating to preventing falls in community-dwelling older people [7] and one focusing on preventing falls in hospitals and aged care facilities [8]. Neither found any RCTs focusing on podiatry-related interventions.

There is evidence that foot problems are associated with increased risk of falls. A recent prospective study of 176 older people indicated that ankle flexibility, toe plantarflexor strength and plantar sensation were significant and independent predictors of balance and functional test performance [6]. A 12-month follow-up of this cohort confirmed that these factors, in addition to foot pain, were significant independent predictors of falls [9].

In addition to foot pain and impairment, inappropriate footwear also plays a role in increasing falls risk [10]. A number of studies have assessed footwear in older people who have fallen, and the evidence indicates that walking barefoot or wearing stockings increases the risk of a fall, as does an increased shoe heel height and smaller sole contact area [11-13]. Furthermore, a number of other studies have investigated the main features of a shoe thought to affect balance, with detrimental effects observed with increased heel height and reduced sole hardness [10, 14-16]. This evidence suggests there is a relationship between footwear and falls and that wearing appropriate footwear may reduce the risk of falls.

Given the emerging evidence that foot problems and inappropriate footwear increase the risk of falls, it has been suggested that podiatry may have a role to play in falls prevention, with several guidelines recommending that older people have their feet and footwear examined by a podiatrist [17, 18].

Several studies have also suggested that some treatments provided by podiatrists, such as lesion debridement [19], foot orthoses [20] and foot and ankle exercises [21], may play a role in improving balance. In addition to the obvious orthotic effect of treatment insoles, an orthotic effect can be achieved through strengthening and activity training programmes as well as through the joint controlling effect of the footwear within which a device is being worn. Apart from one recently completed trial in Australia [22],[23] there has not been a large pragmatic trial looking at the role of podiatric care combined with footwear advice and orthotic inserts to see if this multifaceted intervention can lead to a reduction in falling and is economically viable within the UK health care context. We therefore propose to undertake a randomised controlled trial to evaluate the clinical and cost effectiveness of a multifaceted podiatry intervention for the prevention of falls in patients over the age of 65.

**4.3 Research objectives**

There are three major facets to this protocol, (1) recruitment of a cohort of fallers (2) a pilot study and (3) a randomised controlled trial (RCT).

The first set of objectives (1-6) will be set against the recruitment of the REFORM cohort of fallers and the pilot study. Upon successful achievement of the first six objectives, we will seamlessly progress into a ‘definitive’ RCT (objectives 7-10).

Objectives for the recruitment of an epidemiological cohort and pilot study

1. To demonstrate the feasibility of recruiting to the ‘REFORM’ cohort ie a cohort of people at high risk of sustaining a fall related injury.
2. To develop and pilot a multifaceted podiatry intervention with approximately 60 patients including a foot and ankle exercise programme, supplementary DVD and booklet.
3. To pilot the falls and exercise calendar and other patient data collection questionnaires.
4. To assess participants’ views and experiences of the intervention and the trial process.
5. To develop a podiatrist training package.
6. Pilot, review and refine if necessary recruitment methodology for the main trial.

Objectives for the main REFORM trial

7. To train podiatrists to deliver the intervention.

8. To examine the clinical effectiveness of the multifaceted podiatry intervention for fall prevention.

9. To examine the cost effectiveness of the multifaceted podiatry intervention for fall prevention.

10. To assess podiatrist’s views and experiences of the intervention and the trial process.

**5 Study Design**

The REFORM study will use a “cohort randomised controlled trial (cRCT)” [24] design which will allow us to assemble an epidemiological cohort of men and women over 65 years of age (the REFORM cohort). Key features of the cRCT design are (1) recruitment of a large observational cohort of patients with the condition of interest; (2) regular measurement of outcomes for the whole cohort. This design will enable us to determine the incidence of falls in an older population and observe outcomes among those who do not take part in the trial as well as those who do.

We will first of all recruit the REFORM cohort and once this has been assembled, we will invite a selection of the eligible participants to take part in the REFORM pilot study. Once the pilot study is complete, we will then invite the remaining eligible participants to take part in the REFORM trial. Patients who are ineligible for the REFORM trial will be asked to remain in the cohort and complete follow-up questionnaires. The key phases of the study are shown in the flow diagram in Appendix 1.

**5.1 Recruitment of the REFORM epidemiological cohort**

5.1.1 *Recruitment of sites*

We will recruit ~~1700~~ **2,600** men and women to the REFORM cohort over a 12 month period. Recruitment will take place via NHS podiatry clinics based in either primary or secondary care, falls clinics and GP Practices. Sites will be recruited to the study from the institutions where the co-applicants are based. If additional sites are required, then a member of the study team will provide potential sites with written information and/or visit individual sites to explain the study and what participation would entail.

5.1.2 *Identification of patients to receive invitation mail out*

Patients will be eligible for an invitation mailing if they are male or female and aged over 65 years of age. Sites will be requested where possible to screen out patients in the following groups: patients with a life expectancy of less than six months; patients known to have dementia; patients known to have neuropathy; patients who have had a lower limb amputation; patients known to be chair or bed bound.

All patients who have been identified by the recruiting site as eligible for an invitation mailing will be sent an invitation pack (letter of invitation, participant information sheet, consent form, decline form, screening questionnaire and pre-paid envelope) asking if they would like to participate in the REFORM study. All identifiable information will be held in the NHS until written consent has been obtained from participants.

Where the clinic has the capacity, potential participants may be approached opportunistically. Podiatrists at the clinic or the research podiatrist may either give the patient a REFORM recruitment pack along with verbal information about the study or given the study coordinator's contact details, so that they can contact him/her in person.   Alternatively if the patient prefers (and with their consent) their details may be passed onto the study coordinator who will contact the patient either by phone or send a recruitment pack to them in the post. Where a multidisciplinary team exists other healthcare professionals such as occupational therapists, falls practitioners and physiotherapists may support the opportunistic recruitment. The trial coordinator or podiatry team will provide the HCP with an eligibility check list and support if required.

5.1.3 *Patients who wish to decline participation in the REFORM study*

All patients in the pilot phase of the study who are sent an invitation pack will be given the opportunity to decline participation and if willing, provide some demographic information and reason for declining, in order to provide comparison information with those who are participating. Following the pilot phase of the study we anticipate that we will have collected sufficient information to document the reasons why participants do not wish to take part in the study and will have enough data to be able to compare to those who are participating. The recruitment pack in the main mail out will not contain a decline form. Participants who do not wish to take part in the study, will not have to return any forms to the York Trials Unit.

5.1.4 *Participation in other REFORM studies*

During the consenting stage, potential participants will be informed of the possibility of participating in other related studies eg qualitative study. Consenting participants will be asked to indicate (by ticking a box on the consent form), if they would prefer not to be approached about these studies.

5.1.5 *Patients who wish to take part in the REFORM study*

Patients wishing to take part in the REFORM study will be asked to return their completed consent form and screening questionnaire by post to the York Trials Unit. Researchers at the York Trials Unit will then assess the returned screening forms, for participant eligibility in the study. The participant will be asked to initial each of the statements on the consent form to indicate they agree with them. If however, a participant mistakenly places a tick or a cross in the boxes, these shall be taken as an indication of consent. Nevertheless, all due care will be taken to ensure that the participant initials the boxes as stated on the form.  Any patients who are under 65 years of age, reports having neuropathy, dementia or other neurological condition such as Parkinson’s disease, Alzheimer’s, multiple sclerosis, Lous Gehrig’s or Huntington’s disease, is unable to walk without the help of a walk aid such as a Zimmer frame or walker or person to assist, has had a lower limb amputation, or are unwilling to attend their podiatry clinic will be ineligible to take part in the REFORM study. All eligible, consenting participants will then be asked to complete a baseline questionnaire which may be completed on-line, if the participant prefers. All participants who return valid baseline data will be included in the REFORM cohort. Any participant reporting a score of 10 or more on the Geriatric Depression Scale [25] [26] ie more severe depression, will be referred to their General Practitioner.

Inclusion in the REFORM pilot study and REFORM trial is dependent on participants meeting the eligibility criteria as detailed in section 5.2.1; 5.3.2 and 5.3.3 respectively. Participants who are not eligible will be informed of this outcome and encouraged to complete follow-up questionnaires as part of the REFORM cohort. This cohort will be sent questionnaires at the same time points as participants in the REFORM trial ie monthly falls calendar; six monthly health related quality of life and cost questionnaire post assessment. The questionnaires will be the same as those used for the trial follow-up questionnaires. Participants in the REFORM cohort will be able to withdraw at any point during the study without having to state a reason. If, however the participant indicates the reason for withdrawal, this will be recorded. Data will be retained for all participants up to the date of withdrawal, unless they specifically request that their details are removed.

**5.2 The REFORM pilot study**

The pilot study will consist of:

1. A pilot pragmatic trial evaluating - the designated orthoses as part of a multifaceted intervention with an embedded qualitative study.
2. The development of a multifaceted podiatry intervention including a foot and ankle exercise programme
3. Piloting patient questionnaires and calendars used to collect patient self-reported data
4. To develop a podiatrist training package.
5. Review and, if necessary refinement of recruitment methodology.

5.2.1 Eligibility criteria for the REFORM pilot study

Participants will be eligible for the pilot study if they fulfil all of the following criteria:

1. They are 65 years of age and over
2. Have had either one fall in the past 12 months; or one fall requiring hospital attention in the past 24 months
3. Are willing to participate in the REFORM study
4. The participant is willing to attend the podiatry clinic and receive a podiatric intervention
5. The participant is willing to take part in the qualitative part of the study.

Participants will be excluded from the feasibility study if they fulfil any of the following criteria:

1. If the participant is known to have neuropathy
2. The participant is known to have a neurodegenerative disorder.
3. The participant does not complete the baseline or run-in data collection instruments adequately ie fails to return all monthly falls diaries over a three month period or return baseline questionnaires.
4. If the participant has had a lower limb amputation, or planned lower limb surgery within the three months following the planned initial assessment.
5. If the patient is unable to walk household distances (10 metres) without the help of a walking aid such as a Zimmer frame or walker or person to assist. Patients who use a walking stick however, will be eligible for the study.
6. Patients known to have a life expectancy of less than six months.
7. Patients known to have dementia.
8. Participants are unable to read or speak English.

5.2.2 Recruitment to the pilot study

Participants who have indicated on their REFORM study consent form that they are willing to be contacted about any related studies and who fulfil the eligibility criteria in 5.2.1 will be eligible to be contacted about taking part in the pilot study. Potential participants for the qualitative study will be sent a separate information pack with additional information about the qualitative study which will include an invitation letter, participant information sheet, and a consent form. The qualitative researcher will obtain informed consent from the participant for the qualitative part of feasibility study.

*5.2.2.1 Recruitment and sample size for the pilot study*

In order to explore the feasibility of the intervention, a random sample of 60 participants will be selected from the REFORM cohort. Participants will be allocated equally to each of the two groups ie 30 participants per group. The allocation sequence will be generated by the York Trials Unit randomisation service using a computer and will be stratified by centre and gender.

*5.2.2.2 Recruitment and sample size for the qualitative study*

A purposive sample of 15 participants will be recruited into the qualitative study from the REFORM feasibility study. The selection of these participants will be on the basis of falls history, age and gender. This maximum variation sampling approach [27] will ensure a wide range of viewpoints are included in the data collection and analysis.

5.2.3 Interventions

We will develop a multifaceted podiatry intervention which will consist of the following:

5.2.3.1 Foot orthosis

Because our Australian collaborator has demonstrated that the Formthotics^TM^ orthosis is acceptable to patients and has shown to be associated with a reduction in falls, we have chosen this device for our main trial. However, during the setup of the pilot phase of the study, podiatrists at the recruiting sites commented on the difficulties they had experienced when using Formthotics^TM^  particularly with fitting and posting. Sites frequently use a range of orthotics called the x-line range and frequently use the pressure perfect insole as they are easy to fit in patient’s current shoes. This insole provides basic foot support/control and also has cushioning properties. Where accommodation of the pressure perfect insole in patient’s shoes is difficult, the standard x-line insole from the range may be used. In the pilot phase of the study we will therefore give patients both a Formthotics^TM^  and an x-line insole to wear and take home with them. The x-line orthotic will be fitted and (if indicated) modified to improve foot posture as assessed using the Foot Posture Index and will be modified with prefabricated self-adhesive additions at the discretion of the individual clinician. The Formthotics^TM^  insole will be issued as per the manufacturing guidelines using the appropriate heating equipment and fitting procedure.

5.2.3.2 Foot and ankle exercise programme

The foot and ankle exercise programme, which is currently being used by our Australian collaborator, will be piloted within a UK context and adapted to make it suitable for a UK setting. The exercise programme aims to stretch and strengthen the muscles of the foot and ankle. A summary of the individual exercises which will be used in the home based exercise programme is given in table 1. All exercises will be demonstrated and the patient assessed for competence at the baseline and follow-up appointments. Any patient specific advice will be given with opportunity to contact the podiatrist anytime during the course of the study.

Table 1 Description of home based foot and ankle exercises

| **Activity** | **Description** | **Dosage** | **Increments** |
| --- | --- | --- | --- |
| Ankle range of motion | Sitting with knee extended. Rotate foot in clockwise direction and then anti-clockwise. | 1x10 repetitions for each foot in each direction. | None. |
| Ankle inversion strength | Sitting upright, hip and ankle at 90º. Invert foot against resistive exercise band held by the hand on the same side. | 3x10 repetitions for each foot. | Increase resistance strength of resistive exercise band. |
| Ankle eversion strength | Sitting upright, hip and ankle at 90º. Evert foot against resistive exercise band anchored by the opposing foot. | 3x10 repetitions for each foot. | Increase resistance strength of resistive exercise band. |
| Ankle dorsiflexion strength | Sitting, hip and ankle at 90º.  Dorsiflex both feet to end range of motion and hold. | Hold feet in dorsiflexion for 3x10 seconds. | Increase repetitions up to maximum of 10. |
| Intrinsic strengthening, toe plantarflexion strength and toe stretch | Sitting, hip and ankle at 90 º.  1) Use the therapy ball under the toes to stretch the toes with the foot plantargrade and then curl and point the toes up and over the ball. 2)With the heel on the floor, attempt to pick up the ball with the toes. | 3 x10 repetitions for each exercise both feet. Have a 30 sec break between each repetition | Increase up to a maximum of 50 repetitions. |
| Ankle plantarflexion strength | From standing, rise up onto toes of both feet and then lower back down. | 3x10 repetitions. | Increase repetitions up to maximum of 50. |
| Calf stretch | Sitting, hip at 90 º placing theraband around ball of foot, knee is extended, pull therabands towards body. | Hold stretch for 3x20 seconds on each leg. | Increase forward lean to increase stretch as required. |
| Proprioception/balance training | From standing and holding on to a chair/wall for support, stand on one leg. Repeat on the other side | Hold for 30 seconds.  Repeat 3 repetitions. | Increase slowly to hold for 1 min per repetition.  If competent, rise up on to toes on the one supporting leg: 3 x 10 repetitions. |

5.2.3.3 Footwear assessment and shoe provision

We wish to explore the feasibility of making a footwear assessment, providing footwear advice, provision of the footwear voucher and likely purchase of new footwear. In addition to this we will look at the time taken to explain the exercise programme and fit the device in order to develop the package of care for the main trial.

During this phase of the study we will develop a podiatrist training programme and pilot the patient self-report questionnaires and calendars.

5.2.4.1 Data collection and analysis for quantitative study

We will summarise the overall response rates of follow-up questionnaires categorised by the two intervention groups. Further missing data associated with each question within the questionnaire will be summarised by intervention group. Over all the rates of attrition will be summarised by group.

*5.2.4.2* Nested qualitative study

The qualitative evaluation will be carried out to examine the acceptability of the intervention as a whole package of care to both the trial participants and the podiatry practitioners. Trial participants receiving the intervention will be asked about their experience of receiving the intervention and how they felt this influenced the feasibility study outcome measures - particular attention will be paid to the acceptability and compliance with: the foot orthosis, exercise programme, podiatry service and footwear advice/purchase. We will ascertain from those in receipt of the intervention how this fits into the individual’s wider experience of balance problems within their every day lives. For example, how the intervention fits with their understanding about the reasons for falling, fear of falling, its impact on their life and how they cope with this. We will also ask trial participants more generally about the trial process itself, for example the acceptability of the randomisation procedure and the methods of data collection. This will take the form of established methods for qualitative process evaluation of trials as developed by Donovan et al. [28]

Fifteen trial participants will be purposively selected from the pilot trial. This sample will include 10 participants from the intervention arm and 5 from the control arm and will ensure a maximum variation spread according to age and gender. All participants will be invited to attend a face-to-face interview at a location convenient for them, in order to enhance the rapport between the researcher and the participant and facilitate data collection. However, if the participant preferes the interveiw may be conducted over the phone. The researcher will obtain informed consent from the participant prior to the interview being conducted. The semi-structured interviews will be conducted within approximately two weeks of receiving the 6 month follow-up questionnnaire. All interviews will be conducted using a topic guide to ensure consistency across participants, however, the format will be flexible in order to allow participants to generate naturalistic data on what they constitute as important and/or successful in terms of treatment outcomes and to take into account that only a proportion of the participants have received the intervention. Follow-up telephone interviews will be conducted with all respondents in the two weeks following the 12 month follow-up questionnaires. This will be to discuss any changes (or not) recorded in the outcomes measured over the longer term follow-up period.

We will interview all practitioners who are providing the podiatry services for the study (approximately 5) regarding the way in which they have delivered the intervention/control, the ease of delivery, their confidence in the intervention, and their experience of being involved in the trial. As for the interviews with trial participants, face-to-face or telephone interviews will be conducted using a topic guide and informed consent will be obtained prior to the interview being conducted. Interviews with practitioners will be conducted approximately 3 months into the trial process.

Withdrawal from the qualitative study can occur at any point at the request of the participant. Data will be retained for all participants up to the date of withdrawal, unless they specifically request their details to be removed.

*5.2.4.3 Qualitative analysis*

All interviews will be audio recorded digitally and transcribed verbatim. The computer package ATLAS-ti will be used to manage the data. Data will be analysed according to the constant comparison method through thematic coding of the data. [29] The coding will take place using a combination of a priori themes (according to the aims of the qualitative study and the outcome measures of interest to the trial) and emergent themes. The main focus of the analysis from the pilot phase would be to identify key issues that may inform the process of rolling out into the main trial. However, in addition, as participants in the qualitative sample would also have responses to the quantitative data collected for the feasibility trial, this will allow for the possibility of taking a mixed-methods approach to data integration, in which the two forms of data can be used to complementary way. [30]

**5.3 The REFORM Trial**

Upon successful completion of the pilot stage, the study will progress to a full pragmatic RCT with an economic evaluation.

5.3.1 Trial design

This is a two arm, pragmatic, open randomised cohort controlled trial with a 12 month follow-up.

5.3.2 Inclusion criteria

Participants will be eligible for the REFORM trial if they fulfil all of the following criteria:

1. The participant is 65 years of age and over
2. The participant has had one fall in the past 12 months; or one fall in the past 24 months requiring hospital attention.
3. They are community dwelling.
4. The participant reports a fear of falling i.e. worries about falling all of the time, most of the time, a good bit of the time, some of the time in the past 4 weeks i.e. on their baseline questionnaire.

5.3.3 Exclusion criteria

Participants will be excluded if they fulfil any of the following criteria:

1. The participant is known to have neuropathy.
2. The participant is known to have a neurodegenerative disorder.
3. The participant does not complete the baseline or run-in data collection instruments adequately ie fails to return baseline questionnaires.
4. Participant has had a lower limb amputation
5. The participant is unable to walk household distances (10 metres/32 feet) without the help of a walking aid such as a Zimmer frame or walker or person to assist. Patients who use a walking stick however, will be eligible for the study.
6. The participant is known to have dementia.
7. Participants are unable to read or speak English.

5.3.4. Primary outcome

The primary outcome is the rate of falls (i.e: falls/person/time) where a fall is defined as “an unexpected event in which the participant comes to rest on the ground, floor, or lower level”. [31]

5.3 5 Secondary outcome

Secondary outcomes are:

- Proportion of fallers
- Proportion of multiple fallers (more than two falls)
- Patient reported time to first fall during follow-up
- Health related quality of life as measured by the EQ-5D
- Short Falls Efficacy Scale
- Fear of falling
- Activity of Daily Living
- Fracture rate
- Health service utilisation
- Geriatric Depressions Scale

5.3.6 Sample size

This trial is designed to detect a 10% point reduction in the falls over 12 months. Assuming this high risk group have an underlying risk of 50% (the incidence observed in our recent trial of occupational Therapy for falls reduction) then in order to observe a reduction to 40% with 80% power and a two-sided 5% significance level would require 890 participants (445 in each group, allowing for a 10% loss to follow up). However, we propose to recruit 2,600 participants in to the REFORM cohort to allow us to sample for the feasibility phase and to allow cohort attrition before we sample for the main trial. If more than 890 participants are eligible for the REFORM trial we will recruit these and use unequal allocation, which will allow us to maintain the same size intervention group but increase the size of the control group and increase the power of the study.

5.3.7 Participant withdrawal

Participants can withdraw from the trial at any point by directly contacting the trial coordinator at the York Trials Unit or informing a member of the research team delivering the intervention. If a participant indicates they wish to withdraw from the study, withdrawal will be clarified as to whether the withdrawal is from the intervention only, from follow-up or all aspects of the study. Where withdrawal is only from the intervention then follow-up data will continue to be collected. The reason for the participant wishing to withdraw from the study will not have to be stated however; if the participant indicates the reason this will be recorded. Data will be retained for all participants up to the date of withdrawal, unless the participant specifically requests for their details to be removed.

5.3.8 Randomisation

Participants who fulfil the eligibility criteria and who have provided written consent to take part in the reform study will be eligible for randomisation into the REFORM trial. The randomisation will be carried out by the York Trials Unit Randomisation Service once all relevant data are collected and entered into the study database. Participants will be randomly allocated in a 1:1 ratio to either the intervention or control group. If more than 890 eligible participants are recruited then we will randomly allocate the remaining participants in a 2:1 ratio in favour of the control group. In order to allow for manageable case loads at individual sites, the randomisation will be blocked and stratified by centre with the allocation sequence being computer generated. Participants will be randomised in batches, the frequency of which will be determined by the capacity of the site delivering the intervention. The York Trials Unit (YTU) will write to participants and their GP’s informing them about their group allocation. The podiatrists delivering the intervention will be notified that a new participant has been randomised to the intervention group via email, telephone call or letter. The podiatrist will then access a secure website to retrieve details of the participant and will arrange the required appointments for that participant.

5.3.9 Blinding

Blinding of the participants will not be feasible, nor is blinding of members of the study team who are actively involved in the administration of the study, or the health economist. However, members of study team responsible for the statistical analysis of the study will be kept blind to group allocation.

5.3.10 Intervention group

The intervention group will be seen by the podiatrist as soon as possible after randomisation for an initial visit to assess their foot health. They will receive a multifaceted foot and lower limb intervention. This type of ‘technology’ represents a ‘complex healthcare intervention’ and this study, therefore is informed by the stepwise MRC complex interventions framework. [32] As a result, the pilot trial will address the acceptability and compliance of the complex intervention as a whole package. The multifaceted foot and lower limb intervention will consist of:

1. Footwear advice and provision (where indicated following footwear assessment.)

Participants’ everyday footwear will be assessed using a footwear assessment form which will record the following shoe characteristics; appropriate size; method of fastening; height and width of the shoe’s heel; thickness of outsole; heel counter stiffness, longitudinal sole rigidity; sole flexion point; tread pattern. Footwear will be deemed to be inappropriate if the shoes have any of the following characteristics:

- The heel height is greater than 4.5cm
- No fixation
- No heel counter or the heel counter can be depressed to greater than 45^o^
- A fully worn/smooth/thin sole
- The shoe heel width is narrower than the participant’s heel width by greater than or equal to 20%
- Incorrect shoe size

Participants with inappropriate footwear will be counselled regarding the specific hazardous footwear feature(s) identified during the assessment. The participant will then be advised to purchase a more appropriate pair of shoes. In order to allow participants to do this, they will be given an £80 voucher. This voucher will be redeemable at shoe shops participating in the Healthy Footwear Guide (HFG) Scheme [33] (Current manufacturers participating in the scheme as of March 2011 are: Clarks, DB Shoes, Healthy Feet at [**www.healthyfeet.co.uk**](http://www.healthyfeet.co.uk/), Padders, Simply feet and Websters shoes.) or other shoe shop which the local podiatry clinic would routinely advise patients to purchase shoes from, so this will eliminate the possibility of the participant purchasing non-ideal footwear with the voucher. Alternatively the podiatrists or a member of the research team may order shoes directly from the company. In order to avoid incentivising patients to take part in the study, participants will only be told about the voucher if they are deemed to need new footwear. Recruiting sites who employ a designated ‘shoe fitter’ as part of their routine podiatry service will be asked to accompany patients whilst purchasing new shoes and give advice about the suitability of footwear.

1. Routine podiatry care

Routine podiatry care will be given at a separate Podiatry appointment which will aim to reduce painful conditions, particularly corns and callouses, that has been found to be associated with an increase risk of falls.

1. Foot orthoses:

Participants will be fitted with a prefabricated insole. The type of insole fitted will be determined by the results of the pilot trial and will be either a (Formthotics ^TM^ Foot Science) or an x-line. The orthoses will then be appropriately customised using appropriate self-adhesive additions to improve the foot posture The orthosis will be supplied either by the podiatrist delivering the intervention or by a manufacturer in response to a prescription from the podiatrist.

1. Home based foot and ankle exercise programme

Participants will be prescribed a 30 minute home based foot and ankle exercise programme which should be undertaken three times per week indefinitely. The exercises will be demonstrated by the podiatrist at the participant’s initial visit and will be supplemented by a DVD demonstrating the exercises and an illustrated explanatory booklet showing how to do them at home. The exercise programme which is aimed at stretching and strengthening the muscles of the foot and ankle is based on the programme developed by Spink MJ *et al [22]* and the routine exercises prescribed by podiatrists in the UK.

Participants will complete a questionnaire at 1, 3, 6 and 12 months after randomisation to collect data on compliance with the intervention including the exercise programme footwear advice and insole use, which will be returned to the YTU.

1. Falls prevention leaflet

Participants will receive a copy of the latest falls prevention advice leaflet produced by Age UK. The current version of this document is called “Staying steady. Improving your strength and balance”. This leaflet will be sent to the participant in the post with their baseline questionnaire.

Participants will be sent a site and group specific newsletter at three months and with their six month follow-up questionnaire to inform them about progress with the study. The intervention group’s newsletter will include a section about the foot and ankle exercises in order to aid compliance.

The podiatrist will book a second appointment in approximately two to four weeks time to allow participants sufficient time to purchase new shoes if required. At the second appointment the podiatrist will check the suitability of the new footwear, check that the orthotic device is fitted correctly, customising if necessary. The podiatrist will reinforce the exercise programme. They will ask the participant if they have experienced any adverse events since their last appointment and if they have experienced any problems after undertaking the exercise programme.

It is not anticipated that participants will require a third appointment, however, if participants experience any problems with their orthotic, they will contact the clinic directly to arrange further appointments as required.

5.3.11 Control group

Participants in the control group will receive the same leaflet as the intervention group which will give falls prevention advice. This leaflet will be sent to the participant in the post with their baseline questionnaire. Participants will continue whatever podiatry treatment they currently receive for the 12 months of the study and usual GP care.

Participants will be sent a site and group specific newsletter at three months and with their six month follow-up questionnaire to inform them about progress with the study.

5.3.12 Health economics

The economic analysis will be performed using individual patient level data from the REFORM trial. The analytical approach will take the form of cost-effectiveness and cost-utility analyses. The cost effectiveness approach will assess value for money in terms of per fall averted, and the cost utility analysis will assess cost per quality adjusted life year (QALY) gained. The perspective for both analyses will be that of the UK NHS and Personal Social Service [34] as well as that of the society. Discounting for the future cost and health benefit will not be included considering the time frame for the trial is 12 months after the randomisation. The year of pricing will be set as the mid year of the trial.

Health benefits associated with the treatments will be measured in terms of both estimates of the mean number of falls, corresponding to the main outcome of the trial, and mean QALYs, which is defined as a year lived with full health. The EQ-5D [35] will be used to elicit patient utility values at different points in time and used to calculate QALYs for each patient using the area under the curve. [36] These utility values are used as ‘quality adjustment’ for each patient’s survival time.

Mean within trial estimates of cost and health benefits will be estimated using the regression approach to allow for the correlation between costs and effects as well as adjusting for covariates. This analysis will also account for skewness and censoring associated with time to event and cost data [37] [38] [39]. The result will be presented as incremental cost effectiveness ratio (ICER) where the difference of mean cost estimates between two arms are divided by the different of mean health benefit between two arms.

The uncertainty surrounding the decision to accept a treatment as the most cost-effective will be explored in cost effectiveness acceptability curves (CEAC) [40] These curves depict the probability of accepting a treatment as being cost-effective for a large range of willingness to pay values for an extra unit of health benefit. Sensitivity analysis will be conducted to explore the impact of underlying assumptions of the model and the range of unit costs on the cost effectiveness results.

The main outcome of the trial, fall reduction, could be regarded as an intermediate outcome to achieve the final target - the reduction in fracture. However, due to the restriction in the length of follow-up, the long term effect, the decreasing number of fractures, might not be observed in the current trial. Therefore a further analysis here is to explore the possible long term impact of the trial assuming that a falls reduction should also lead to a fracture reduction.

A decision analytic model approach will be adopted to perform such a task. The perspective will be the UK NHS and Personal Social Service and time horizon for this analysis will be a life-time horizon. Life-time horizon refers to following up every single participant in a hypothetical cohort until the last participant dies. The hypothetical cohort will be constructed, based on the characteristics of the trial population, to estimate the QALY yield and cost saving of the long term effect of the intervention. The model parameters which are not collected in the trial will be extracted from the existing literature. The model outputs will be the estimated expected mean costs, effectiveness, and QALYs associated with each alternative treatment. Estimated total costs and outcomes will be discounted properly according to the latest guidance of health technology appraisal.

Uncertainty regarding cost-effectiveness will be evaluated using probabilistic sensitivity analysis, where inputs into the analysis are defined as probability distributions which reflect uncertainty. The uncertainty surrounding the decision to adopt a given treatment option as a cost-effective treatment as different levels of willingness to pay will be represented in acceptability curves. The impact of assumptions undertaken in the analysis regarding the evidence over parameters or relating to the decision model (such as extrapolation) will be evaluated in sensitivity analysis, if possible

5.3.13 Adverse events

Details of any adverse events reported to the York Trials Unit either directly by the participant or by a member of the research team at the recruiting site will be recorded using the appropriate adverse event form and will be stored in the participant’s records.

This study will record details of any serious adverse events (SAEs) that are required to be reported to the Research Ethics Committee (REC) under the current terms of the Standard Operating Procedures for RECs. Non-serious adverse events will not be recorded or reported for this study.

A Serious Adverse Event (SAE) is defined as any untoward occurrence that:

(a) Results in death;

(b) Is life threatening;

(c) Requires hospitalisation or prolongation of existing hospitalisation;

(d) Results in persistent or significant disability or incapacity;

(e) Consists of a congenital anomaly or birth defect; or

(f) Is otherwise considered medically significant by the investigator.

An event is defined as ‘related’ if the event was due to the administration of any research

procedure. Whereas an ‘unexpected event’ is defined as: a type of event not listed in the protocol as an expected occurrence.

In the context of this study, an occurrence of the type listed in (a) to (f) will be reported as an SAE only if:

- The event is suspected to be related to an aspect of the research procedures

(e.g. wearing the orthotic, undertaking the exercise programme, completion of follow-up questionnaires, participation in feasibility or qualitative sub-studies, telephone contact).

AND

- It is an unexpected occurrence.

Hospitalisations, disabling / incapacitating / life-threatening conditions, falls and deaths are expected in the study population due to the age of the cohort, they will therefore only be reported as SAEs if they appear to be related to an aspect of taking part in the study. Expected adverse events which are related to the intervention include: aches and pains in the lower limb for longer than 48 hours; fall; new callus/corn formation, blisters, ulcers; skin irritation/injury including pressure sores; soft tissue injury. If a participant or a member of the research team rings the York Trials Unit to notify the occurrence of an adverse event, or an adverse event is reported in information provided in the follow-up questionnaires, then the trial coordinator – or designated person if unavailable should be informed immediately by telephone, email or in person. The trial coordinator (or designated person) will inform the Chief Investigator (CI) or designated person, and two members of the Trial Management Group (TMG) who will jointly decide if the event should be reported to the main REC as an SAE. Related and unexpected SAEs will be reported to the main REC within 15 days of the CI becoming aware of the event. Details of the assessment will be recorded on an ‘adverse event review form’.

The occurrence of adverse events during the trial will be monitored by an independent Data Monitoring Ethics Committee (DMEC)/ Trial Steering Committee (TSC). The DMEC/TSC will immediately see all SAEs thought to be treatment related and they will see SAEs not thought to be treatment related by the Trial Management Group at the next scheduled meeting.

**5.4 Data collection and analysis**

5.4.1 Quantitative data collection

Participant self-reported data will be collected at the following time points: at invitation, baseline (pre-randomisation/pre-assessment), monthly falls calendars and at six and 12 months post-randomisation/post-assessment for trial and cohort participants. Data on compliance to the intervention will be collected via questionnaire sent at 1, 3 6 and 12 months. For data collected post-randomisation/post assessment an option to complete the data on-line will be offered.

Follow-up questionnaires will be sent to participants in the post by the research team based at the University of York’s Trials Unit. Participants who provide an email address will be sent a pre-notification email, two weeks prior to the questionnaire being sent in the post. This email will alert participants that they will soon receive a follow-up questionnaire and that they can, if they prefer, complete the questionnaire on-line. Participants who do not return their follow-up questionnaire within two weeks will be sent up to two reminders, two weeks apart either by post, text or email. Members of the research team may also contact participants by telephone, post or email or text as per the participant’s preference regarding any queries they may have in relation to the follow-up questionnaires, exercise diaries or falls calendars. All participants will be sent an unconditional £5 with the 12 month questionnaire in recognition of their commitment to the study and to cover any expenses incurred in completing the questionnaires. When a participant has been lost to follow-up, their data will be included in the main analysis up to where they have been lost to follow-up.

Process data collected via the podiatrists will be collected at the patient’s initial and follow-up attendance to the podiatry clinic. Additional data will be collected if the participant attends additional trial appointments. Attendance to the podiatry clinic by the control participants and details of the treatment/advice given will be recorded.

5.4.2 Data collection for the primary outcome for the REFORM Trial

The primary outcome is the rate of falls (ie falls/person/time). A fall is defined as “an unexpected event in which the participant comes to rest on the ground, floor, or lower level”. [31] Data will be collected via participant self-reported falls calendars in the 12 months following randomisation. Participants will be asked to record if they had any fall or not for each day. Participants who do not return their falls calendar within one week of the due date will be telephoned by the YTU, and information collected from the patient over the phone. Participants will also be given the YTU free phone number to ring during office hours to report any fall they have as soon as possible after the fall. The YTU personnel will collect information including: details of the event ie date of event, cause/reason for fall, consequence of fall: superficial wound (bruising, sprain, cut, abrasions) or fractures and type of fracture, footwear worn whilst having the fall, hospital admissions, if the patient was wearing their orthotic at the time of falling or if they were undertaking the prescribed exercises, EQ5D. However, as we are also collecting falls data from the six and 12 month follow up questionnaires, these data will be used for those participants to do not return their monthly falls calendar.

5.4.3 Data collection for secondary outcomes and process data for the REFORM Trial

Patient self-reported data from monthly falls calendars, monthly exercise calendars and follow-up questionnaires will be used to capture the following data:

- Proportion of fallers
- Proportion of multiple fallers
- Proportion of single fallers
- Participant self-reported time to first fall during follow-up over the 12 month follow-up period
- Health related quality of life as measured by the EQ-5D
- Short Falls Efficacy Scale
- Fear of falling
- Activity of Daily Living
- Fracture rate
- Health service utilisation data
- Geriatric depressions scale

5.4.4 Statistical analysis

There will be one single analysis at the end of the trial. All analysis will be conducted using STATA, (StataCorp, version 10.1, 4905 Lakeway Drive, College Station, Texas 77845 USA) and SAS (SAS Proprietary Software version 9.2, SAS Institute Inc., Cary, NC, USA) All analyses will be conducted on an intention to treat basis, ie including all randomised patients in the groups to which they were assigned. If more than 890 participants are recruited and unequal allocation is utilised then analyses will be adjusted to take this into account. The trial statistician and health economist will write detailed analysis plans, based on the protocol after it has been approved.

5.4.5 Statistical analysis of the primary outcome

The number of falls per person will be analysed using a Poisson regression model adjusting for gender, age and history of falling to estimate the difference in falls rate between the groups. If there is over dispersion a negative binomial regression model adjusting for the same factors will be used [41] Point estimates and their associated 95% confidence intervals will be provided. For the main outcome a significance level of 0.05 will be used.

5.4.6 Statistical analysis of secondary outcomes

The proportion of fallers versus non-fallers in each group will be compared by logistic regression adjusting for gender, age, centre and history of falling. Odds ratios and their associated 95% confidence intervals will be provided.

The proportion of multiple fallers versus single or non-fallers in each group will be compared over the 12 month trial period using logistic regression adjusting for gender, age, centre and history of falling.

The time to the first fall will be derived as the number of days from randomisation until the patient reports having a fall as detailed from the participant’s falls calendar, telephone notification form or self-reported questionnaire. Participants who have not had a fall will be treated as censored at their date of trial exit, or date of last available assessment or 365 days/trial cessation, as appropriate.

The proportion of patients yet to experience a fall between the two groups will be summarised by a Kaplan Meier survival curve. The risk of experiencing a fall between the two groups during follow up will be analysed by Cox Proportional Hazard regression adjusting for gender, age, centre and history of falling. Hazard ratios and their associated 95% confidence intervals will be provided. The proportional hazard assumption will be evaluated using Schoenfeld residuals. The median time to the first fall and its associated 95% confidence intervals will be estimated from this adjusted model.

The proportion of people with depression in each group will be compared over the 12 month trial period using logistic regression adjusting for gender, age, centre and history of falling.

The following secondary outcomes, Short Fall Efficacy Scale, Fear of falling and Activity of Daily living, Geriatric Depression Scale are continuous and will be measured at baseline, month 6 and month 12. Linear mixed models will be used to compare the two groups on these outcomes adjusting for gender, age and history of falling. Random intercepts for each patient will be included in the models. Model effects and their associated 95% confidence intervals will be provided.

The secondary outcome fracture (yes/no) will be measured at baseline, month 6 and month 12. The fracture rate between the two groups will be compared using correlated logistic regression estimated using Generalised Estimating Equations (GEE) with robust standard errors adjusting for gender, age and history of falling . Model effects and their associated 95% confidence intervals will be provided

Missing data and sensitivity analyses

Missing data will be assumed to be Missing At Random (MAR) in the aforementioned statistical methods. We will therefore investigate departures from this assumption in a sensitivity analysis using pattern mixture models and selection models.

Analysis of cohort group

Descriptive statistics will be presented for the cohort group.

5.4.7 Intervention adherence

The number of participants in the intervention group adhering to the exercise and orthotic component of the intervention will be collected from participant self-reported questionnaire data at 1, 3, 6 and 12 months. Participants will be asked to record if they were wearing their orthotic ‘all of the time’, ‘most of the time’, ‘some of the time’, ‘a little of the time’ or ‘none of the time’. Adherence to the exercise programme by the intervention group will be measured by participant self-reported completion of the exercise diaries. Descriptive statistics will be used to describe the proportion of self-reported completion of the exercise programme and the percentage of participants completing three exercise sessions per week over 1, 3, 6 and 12 month period.

Similarly the adherence of participants wearing new shoes as part of the prescribed intervention will be measured; those previously assessed as having inappropriate footwear, whom were given advice and received a footwear voucher to purchase appropriate shoes. Descriptive statistics will be used to describe the compliance of these participants for each aspect of this part of the intervention.

A Complier Average Causal Effect (CACE) analysis to assess the impact of compliance on treatment estimates will be undertaken.

5.5 Definition of the end of the trial

The end of the REFORM trial is defined as the date when the NIHR HTA Programme trial report is due.

1. **Trial monitoring**

6.1 Site monitoring

Site monitoring visits for this study will not be undertaken on behalf of the sponsors since (a) eligibility for the study is undertaken by review of patient’s self-reported data sent to the York trials unit (b) the majority source data for this study is patient self-report questionnaires which is returned directly to the York Trials Unit and (c) data on adverse events will mainly be collected via patient self-reported data to the York Trials Unit. Participating sites will, however, be asked to assist in trial-related monitoring when required for example for audits, ethics committee review and regulatory inspections.

6.2 Standard operating procedures

This trial will be run in accordance with the University of York, Dpt Health Sciences York Trials Unit’s Standard Operating Procedures.

1. **Ethical issues**

We are aware that some older people may represent a vulnerable group. However, we do not anticipate any major ethical issues with this study. Participation in the study is voluntary, those patients who are approached to take part in the study and who return their decline form to the York Trials Unit, will not be contacted by the research team again. Participants will not be denied any form of care that is currently available in the NHS by participating in the trial, subject to local provision of services. Participants will be able to withdraw from the study at any point by contacting the trial coordinator or the podiatrist delivering the intervention.

- 1. Anticipated risks and benefits

The study does not involve any invasive/potentially harmful procedures and is therefore considered low risk for participants. The trial intervention consists of a multifaceted foot and lower limb intervention. The orthotic component does not involve a new medical device, and all orthotics used in the trial will be CE marked. Patients who would otherwise be disadvantaged by using the trial insole will either be advised to continue wearing their current insole prescription (if already in place) or will be referred for a more specialist insole to address their more complex clinical need. The home based foot and ankle exercise programme has been developed by podiatrists based in Australia, one of whom is a grant applicant on the study [22] and modified to reflect current routine practice in the UK. It has been used without incident, as part of a multifaceted podiatry intervention in a trial aimed at preventing falls in patients over 65 years. On demonstration of the exercise the individual patient will be assessed and the programme altered accordingly to their specific needs.

- 1. Informing participants of anticipated risks and benefits

The patient information sheet will provide potential participants with information about the possible benefits and anticipated risks of taking part in the study either as a participant in the cohort or additionally in the trial. Participants will be given the opportunity to discuss participation with the trial coordinator prior to consenting to participate. The trial coordinator will inform the participant if new information comes to light that may affect the participant’s willingness to participate in the study.

- 1. Obtaining consent

Participation in the study will be entirely voluntary. Potential participants will receive an information pack about the trial. The pack will contain an invitation letter, patient information sheet, a consent and a decline form, screening questionnaire and pre-paid envelope. The patient information sheet will be produced using the current guidelines for researchers on writing information sheets and consent forms, posted on the NRES website. Potential participants will be given the trial coordinator’s telephone number to phone if they have any queries about taking part in the study. The qualitative researcher will obtain informed consent from the participant for the qualitative part of the study.

- 1. Retention of study documentation

All data will be stored for a minimum of five years after the end of the main analysis of the trial. All paper records will be stored in secure storage facilities. Personal identifiable paper records will be stored separately from anonymised paper records. All electronic records will be stored on a password protected server within the York Trials Unit.

1. **Service User Involvement**

We will establish a patient reference group (PRG), consisting of volunteers who have balance problems or a history of falls. The PRG will be recruited from recruitment sites and through contact with patient groups (eg. Age Concern).

The PRG will comprise approximately four people and will meet regularly during the research process. Particular emphasis will be given to the participation of the PRG during the feasibility phase where their input will complement the data on acceptability and comfort generated from the qualitative phase of the project.

A member of the PRG will be asked to co-chair the meetings (supported by the Chief Investigator) to ensure that there is a two way communication between the PRG and the research team. At least one member of the PRG will be asked to join the Trial Steering Group.

The PRG meetings will be attended by the Chief Investigator and at least two co-applicants and researchers on the project. The PRG will be given minutes of all the research team meetings and be asked to provide input to all elements of the research study, including the design of questionnaires, topics to be included in the topic guide for the qualitative interviews and trial methods.

The PRG will also be essential in assisting and reviewing all patient information, including the participant information sheets and informed consent. Minutes of the PRG meetings will be reported on during the research team meetings. The PRG will also be asked to comment on any dissemination activity that results from the study.

1. **Trial Management**

9.1 Sponsorship

The University of York will act as the sponsor for the REFORM study.

Contact details: Mrs Sue Final

Intellectual Property Manager

University of York

Enterprise and Innovation Office

Innovation Centre

York Science Park

York

YO10 5DD

9.2 Indemnity

NHS Indemnity will apply for patients treated within NHS sites. The University of York will provide legal liability cover for their employed staff. Non-negligent harm will not be covered.

9.3 Funding

Research funding has been secured from the National Institute of Health Research – Health Technology Assessment programme (reference: 09/77/01)

The main cost implication for the NHS is podiatrist time. This is classed as a Service Support Cost. From 2008/2009 service support costs have been met via the UKCRN Clinical Research Network Portfolio. It is anticipated that service support costs for REFORM will be met via this route once it has been adopted as a portfolio study.

- 1. Independent Steering Committee

Due to the low risk nature of this trial, approval will be sought from the funders to set up one independent steering and monitoring committee to undertake the roles traditionally undertaken by the Trial Steering Committee (TSC) and Data Monitoring and Ethics Committee (DMEC). This committee will comprise of an independent chair who will be a clinician with expertise in falls prevention, a statistician, a podiatrist, the chief investigator and other study collaborators. The independent members of the committee will be allowed to see unblinded data, but unblinded data would not be reported to the other members of the research team. The role of this committee will include the review of all serious adverse events which are thought to be treatment related and unexpected. The committee will meet at least annually or more often as appropriate. If however, the funders do not agree to one overarching committee, separate TSC and DMEC will be set up. The TSC will include an independent chair and at least two other independent members, along with the chief investigator and the other study collaborators. The DMEC will comprise of an independent chair, a statistician and a podiatrist. Both committees will meet annually. The role of the DMEC will be to immediately see all serious adverse events which are thought to be treatment related and unexpected.

9.5 Trials management group (TMG)

A TMG will be formed. It will consist of the Chief Investigator; (who will be in charge of the overall management of the study), a trial coordinator; (who will be responsible for the day-to-day management of the study); the study’s grant co-applicants; the research podiatrist and the principal investigators at sites delivering the intervention. Regular meetings will be held according to the needs of the trial.

1. **Publication Policy**

It is intended that the results of the study will be reported and disseminated in peer-reviewed scientific journals. The funders, the NIHR HTA currently publish all research reports on their website <http://www.hta.ac.uk/project/htapubs.asp> and it is anticipated that the full trial report will be available approximately a year after the report is submitted to the funders. We will also aim to publish in professional journals to ensure that healthcare professionals have prompt access to the study’s findings. The results of the study will be submitted for consideration at the Society of Chiropodists and Podiatrists Annual Conference. We will produce a short summary of the results of the study which will be distributed to all trial participants.

**Appendix 1 Recruitment to the REFORM cohort and the REFORM pilot trial**

**Intervention Group**

**N = 30**

Intervention developed and podiatrist training package developed

**REFORM cohort recruitment: patients consenting to follow up are sent a baseline questionnaire, monthly falls calendars; 6 monthly QoL questionnaires. Data collection continues for 12 months if patient ineligible for the REROEM trial.**

Patients over 65 are sent an invitation letter, patient information sheet, consent/decline form, screening questionnaire inviting them to take part in the REFORM study to approximately 1,500 potential participants

**Control Group**

**N = 30**

Baseline data*

Falls prevention advice

Routine podiatry

Orthotic assessment

Footwear advice

Foot and ankle exercises

Baseline data*

Falls prevention advice

6 month follow-up*

Qualitative data collection

12 month follow-up*

Qualitative data collection

6 month follow-up*

Qualitative data collection

12 month follow-up* Qualitative data collection

2-4 weeks Clinical review

Orthotic fitting

**Monthly falls diary***

Eligible members of the cohort are randomised to the pilot phase of the study.

Consenting, eligible participants are sent a baseline questionnaire.

**Monthly falls diary; 1, 3, 6 and 12 month compliance data questionnaire , 6an***

3 month qualitative data collection from podiatrists

*Piloting of trial documentation

**The REFORM trial**

Members of the cohort who are compliant with follow-up and still willing to participate

**Intervention Group**

**N = 445**

**Control Group**

**N = 445**

Baseline data

Falls prevention advice

Routine podiatry

Orthotic assessment

Footwear advice

Foot and ankle exercises

Baseline data

Falls prevention advice

6 month follow-up

12 month follow-up

6 month follow-up

12 month follow-up

Clinical review

Orthotic fitting

**Monthly falls diary**

**Monthly falls diary**

1 month compliance data

3 month compliance data

Appendix 2: Study timeline

REFORM study – overview of phased approach and timeline

Ethics, Recruitment of REFORM TRIAL Follow –up Analysis and

Research REFORM cohort; Clinical & cost effectiveness evaluation write up Governance, REFORM pilot study REFORM qualitative study (Podiatrists)

Portfolio REFORM qualitative study (Patients)

Adoption

Time in T – 6 0 18 24 36 42

months

Trial coordinator (42 months @1.0 FTE)

Trial secretary (42 months @0.5 FTE)

Trial statistician (42 months@ 0.18 FTE)

Data management (42 months @ 0.25 FTE x 2

Research podiatrist (36 months @ 0.5 FTE)

Appendix 3: Data collection schedule

|  | Invitation | Baseline | 2/3 week follow-up | Monthly data collection | 1 & 3  months | 6 month follow-up | 12 month follow-up |
| --- | --- | --- | --- | --- | --- | --- | --- |
| Consent/Decline form | √ |  |  |  |  |  |  |
| Demographic questionnaire | √ | √ |  |  |  |  |  |
| Falls questions | √ | √ |  |  |  | √ | √ |
| Falls calendars |  |  |  | √ |  | √ | √ |
| Geriatric Depression score |  | √ |  |  |  | √ | √ |
| Resilience questions |  | √ |  |  |  | √ | √ |
| EQ-5D |  | √ |  |  |  | √ | √ |
| Short falls efficacy scale |  | √ |  |  |  | √ | √ |
| Fear of falling |  | √ |  |  |  | √ | √ |
| Activity of Daily Living |  | √ |  |  |  | √ | √ |
| Fracture data |  | √ |  |  |  | √ | √ |
| Economic evaluation |  | √ |  |  |  | √ | √ |
| Footwear assessment & advice* |  | √ | √ |  |  |  |  |
| Exercise demonstration and advice * |  | √ | √ |  |  |  |  |
| Foot posture index* |  | √ |  |  |  |  |  |
| Foot problems* |  | √ |  |  |  |  |  |
| Exercise footwear and orthotic compliance calendars* |  | √ |  | √ | √ | √ | √ |
| Orthotic prescription/review* |  | √ |  |  |  |  |  |
| Routine podiatry care (will continue outside of trial time as routine)* |  | √ | √ |  |  |  |  |

*Intervention group only

REFERENCES

1. Iglesias, C., Manca, A., and Torgerson, D. *The health-related quality of life and cost implications of falls in elderly women.* Osteoporos Int (2009) (20): p. 869–878 DOI 10.1007/s00198-008-0753-5.

2. Lord, SR., Sherrington, C., Menz, HB., et al*. Falls in older people: risk factors and strategies for prevention*. 2007: Cambridge University Press, Cambridge.

3. Dunn, J., Link, CL., Felson, DT., et al*.* *Prevalence of foot and ankle conditions in a multiethnic community sample of older adults.*  American Journal of Epidemiology 2004. 159: p. 491-498.

4. Benvenuti, F., Ferrucci, L., Guralnik, JM., et al. *Foot pain and disability in older persons: an epidemiologic survey.* Journal of the American Geriatrics Society 1995. 43: p. 479-484.

5. Barr, E., Browning, C., Lord, SR., et al. *Foot and leg problems are important determinants of functional status in community dwelling older people.* Disability and Rehabilitation 2005. 27: p. 917-923.

6. Menz, H., Morris, M., and Lord, S. *Foot and ankle characteristics associated with impaired balance and functional ability in older people.* Journal of Gerontology 2005. 60A: p. 1546-1552.

7. Gillespie, L., Robertson, MC., Gillespie, WJ., et al. *Interventions for preventing falls in older people living in the community.* Cochrane Database Syst Rev 2009;2:CD007146.

8. Cameron, I., Murray, GR., Gillespie, LD., et al., *Interventions for preventing falls in older people in nursing care facilities and hospitals .* Cochrane Database Syst Rev 2010: p. 1:CD005465.

9. Menz, H., Morris, M., and S. Lord, S. *Foot and ankle risk factors for falls in older people: a prospective study.* Journal of Gerontology 2006**.** 61A**:** p. M866-870.

10. Menant, J., Steele, JR., Menz, HB., et al. *Rapid gait termination: effects of age, walking surfaces and footwear characteristics.* Gait Posture, 2009. 30: p. 65-70.

11. Menz, H., Morris, M., and Lord, S. *Footwear characteristics and risk of indoor and outdoor falls in older people.*  Gerontology 2006. **52**: p. 174-180.

12. Tencer, A., Koepsell TD., Wolf ME. et al. *Fall-risk screening test: A prospective study on predictors for falls in community-dwelling elderly.* Journal of Clinical Epidemiology 2001. 44: p. 837-844.

13. Koepsell, T., Wolf ME., Buchner DM. et al. *Footwear style and risk of falls in older adults .* Journal of American Geriatriatric Society 2004. 52: p. 1495-1501.

14. Menant, J., Steele JR., Menz HB. et al. *Optimizing footwear for older people at risk of falls.* Journal Rehabilitation Research Development 2008. 45(1167-81).

15. Menant, J., Steele JR., Menz HB., et al. *Effects of footwear features on balance and stepping in older people.* Gerontology 2008. 54: p. 18-23.

16. Lord, S. and G. Bashford, *Shoe characteristics and balance in older women.* Journal of the American Geriatrics Society 1996. 44: p. 429-433.

17. American Geriatrics Society and BGSaAAoOS. *Guideline for the prevention of falls in older persons.*  Journal of the American Geriatrics Society 2001. 49: p. 664-672.

18. Australian Commission on Safety and Quality in Healthcare. *Preventing falls and harm from falls in older people: best practice guidelines for Australian community care. Commonwealth of Australia.* 2009.

19. Balanowski, K. and Flynn, L.  *Effect of painful keratoses debridement on foot pain, balance and function in older adults.* Gait and Posture 2005. 22: p. 302-307.

20. Hijmans, J., Geertzen, JHB, Dijkstra, PU., et al. *Systematic review of the effects of shoes and other ankle or foot appliances on balance in older people and people with peripheral nervous system disorders.* Gait and Posture 2007. 25: p. 316-323.

21. Kobayashi, R., Hosoda, M., Minematsu, A., et al. *Effects of toe grasp training for the aged on spontaneous postural sway.*  Journal of Physical Therapy Science 1999. 11: p. 31-34.

22. Spink, M., Menz, HB., and S. Lord, S. *Efficacy of a multifaceted podiatry intervention to improve balance and prevent falls in older people: study protocol for a randomised trial* BioMed Central Geriatrics 2008. 8: p. 30.

23. Spink, MJ., Menz, HB., Fotoohabadi, M.R., et al. *Effectiveness of a multifaceted podiatry intervention to prevent falls in community dwelling older people with disabling foot pain: randomised controlled trial.*  British Medical Journal, 2011. 342:d3411doi:10.1136/bmj.d3411.

24. Relton, C., Torgerson, DJ., O’Cathain, A., et al. *Rethinking pragmatic randomised controlled trials: introducing the "cohort multiple randomised controlled trial" design.* British Medical Journal, 2010. 340:Mar 19;340:c1066. doi: 10.1136/bmj.c.1066.

25. Brink TL, Yesavage, JA., Lum, O., et al. *Screening tests for geriatric depression.* Clinical Gerontologist 1982. 1( 37-44).

26. Sheikh, JI., and Yesavage, JA. *Geriatric Depression Scale (GDS): Recent evidence and development of a shorter version. A Guide to Assessment and Intervention*. Clinical Gerontology. Vol. 165-173, . 1986: The Haworth Press. 165-173.

27. Patton, M. *Qualitative Evaluation and Research Methods* 2nd ed ed. 1990 Newbury Park, CA: Sage.

28. Donovan, J., Mills, N., Smith, M., et al. *Improving design and conduct of randomised trials by embedding them in qualitative research: ProtecT (prostate testing for cancer and treatment) study.* Britiash Medical Journal. 2002. 325: p. 766-770.

29. Silverman, D. *Interpreting Qualitative Data: Methods for Analyzing Talk, Text and Interaction.* 1993 London: Sage

30. Adamson, J. *Combined qualitative and quantitative designs. In: Bowling A, Ebrahim S, editors. Handbook of Health Research Methods.Maidenhead.*  2005: Open University Press.

31. Lamb, S., Jorstad-Stein, EC., Hauer, K., et al. *Development of a common outcome data set for fall injury prevention trials: the Prevention of Falls Network Europe consensus.*  Journal American Geriatric Society. 2005. 53(9): p. 1618-22.

32. Campbell, M., Fitzpatrick, R., Haines, A., et al. *Framework for design and evaluation of complex interventions to improve health.*  British Medical Journal. 2000. 321: p. 694-6.

33. Vernon, W. *The Healthy Footwear Guide" .* Podiatry Now. 2009. 12 (5, May ): p. 35.

34. Excellence., N.I.f.H.a.C., *Guide to the Methods of Technology Appraisal. London, UK: National Institute for Health and Clinical Excellence*. 2008.

35. Brook, R. *EuroQol: the current state of play.*  Health Policy, 1996. 37: p. 53–72.

36. Matthews, J., Altman, D., Campbell, MJ. *Analysis of serial measurements in medical research.*  British Medical Journal 1990. 300: p. 230-235.

37. Nixon, RM., Thompson, SG. *Methods for incorporating covariate adjustment, subgroup analysis and between-centre differences into cost-effectiveness evaluations. .* Health Econ 2005. 14: p. 1217-1229.

38. O'Hagan, A., Stevens, J., Montmartin, J. *Bayesian cost-effectiveness analysis from clinical trial data.*  Statistics in Medicine 2001. 20: p. 733-753.

39. Willan, A., Lin, D., Manca, A. *Regression methods for cost-effectiveness analysis with censored data.* Statistics in Medicine 2005. 24: p. 131-145.

40. Van Hout BA, Al, MJ., . Gordon, GS., et al. *Costs, effects and c/e-ratios alongside a clinical trial.*  Health Economics, 1994. 3: p. 309-319.

41. Robertson, M., Campbell, A., Herbison, P. *Statistical Analysis of Efficacy in Falls Prevention Trials* Journal of Gerontology, 2005. 60A(4 ): p. 530–534.
